# PROTOCOL: Non‐criminal justice interventions for countering cognitive and behavioural radicalisation amongst children and adolescents: A systematic review of effectiveness and implementation

**DOI:** 10.1002/cl2.70020

**Published:** 2025-01-15

**Authors:** James Lewis, Sarah Marsden, Anna Stefaniak, James Hewitt

**Affiliations:** ^1^ School of International Relations, Handa Centre for the Study of Terrorism and Political Violence (CSTPV) University of St Andrews St Andrews Fife Scotland; ^2^ School of Psychology and Neuroscience University of St Andrews St Andrews Fife Scotland

**Keywords:** children, countering violent extremism, evaluation, implementation, interventions, non‐criminal justice, youth

## Abstract

This is the protocol for a Campbell systematic review. The objectives are as follows. (1) Examine whether secondary and tertiary interventions delivered outside of the criminal justice system are effective at countering the cognitive and behavioural radicalisation of children and adolescents by synthesising evidence relating to relevant primary and secondary outcomes of effectiveness. (2) Examine whether secondary and tertiary interventions delivered outside of the criminal justice system are being implemented as intended by synthesising evidence that captures how interventions are implemented, considering whether they are implemented as expected or in ways that align with their underlying logic. (3) Identify those implementation factors (facilitators and barriers) and moderators that impact how interventions working with children and adolescents are delivered.

## 1 BACKGROUND

This review examines the pressing issue of youth radicalisation, and current efforts to counter the radicalisation of children and adolescents (i.e., those aged 19 years or under) outside of the criminal justice system. It examines the effectiveness and implementation of interventions working with children and adolescents in this context and identifies relevant lessons for research and practice.

### 1.1 The problem, condition or issue

The concept of radicalisation first emerged in counterterrorism policy circles at the start of the 21st Century (Neumann, 2013). It has been, and remains, a contested concept, with some authors challenging the evidence base underpinning dominant models of radicalisation, and the term's lack of conceptual clarity (Ford & Jackson, 2023; Richards, 2015). Nevertheless, the concept has become embedded in counterterrorism policy around the world and has come to guide efforts to prevent and counter violent extremism (P/CVE) in the Global North and South (Lewis, Marsden, Cherney, et al., 2024).

There is no single definition of radicalisation. However, many definitions identify radicalisation as having both cognitive and behavioural dimensions (Neumann, 2013; Vergani et al., 2020). For example, the US Department of Homeland Security (Department of Homeland Security [DHS], 2017) defines radicalisation as ‘[t]he process through which an individual changes from a non‐violent belief system to a belief system that includes the willingness to actively advocate, facilitate, or use unlawful violence as a method to effect societal or political change’. In adopting this definition, we understand radicalisation as producing cognitive and behavioural outcomes and delineate between cognitive and behavioural forms of radicalisation. As defined by Vergani et al. (2020, p. 859) cognitive radicalisation is a process resulting in ‘support for violent extremist acts (e.g., terrorist attacks), people (e.g., Anders Breivik), and groups (e.g., Al Qaeda) that committed acts of violent extremism (e.g., terrorism)’; whereas behavioural radicalisation results in an individual ‘committing an act of violent extremism (e.g., terrorism) or joining a violent extremist group (e.g., Islamic State of Iraq and the Levant or Al Qaeda)’.

Youth radicalisation has become a pressing concern for governments around the world (Bronsard et al., 2022). There has been a notable recent increase in the number of children and adolescents – defined by the World Health Organisation (WHO, undated) as people aged 19 years or under – who are coming into contact with the counterterrorism system in different countries (e.g., Barracosa & March, 2022; Bronsard et al., 2022; Rose & Vale, 2023). This trend is evident across most areas of counterterrorism policy and practice, ranging from the preventive space, where children and adolescents now make up a significant proportion of the caseloads of CVE interventions working with individuals assessed as being at risk of radicalisation (see Section 3.1.2) or at an early stage of radicalisation (e.g., Bronsard et al., 2022; Home Office, 2023), through to the correctional context, where several countries have seen a recent increase in the numbers of children and adolescents convicted for terrorism offences (e.g., Europol, 2022; Renwick, 2018; Rose & Vale, 2023).

There is no single pathway or process of radicalisation (Horgan, 2008). Instead, research highlights that processes of radicalisation are marked by the intersection of different individual, familial, social, and environmental factors that are specific to individual cases and contexts (Koehler, 2020). Processes of youth radicalisation are no different in this regard, and a number of youth‐specific models of radicalisation developed in recent years point to this intersection of factors operating at different levels of analysis (e.g., Campelo et al., 2018). However, research is increasingly pointing to distinctions between processes of youth and adult radicalisation. These analyses discuss, for example, the relevance of different risk and protective factors in the radicalisation of youth when compared to adults (Oppetit et al., 2019); the particular role of the internet in youth radicalisation (Cherney, Belton, et al., 2022); and how the context of childhood and adolescence, which are key periods of development and transition, might inform radicalisation processes (Beelmann, 2021). The complexity and distinctiveness of youth radicalisation therefore poses specific challenges for policy.

Concerns about childhood and adolescent radicalisation have precipitated notable developments in policy and practice. Government agencies have increasingly looked to the criminal justice system to tackle this issue, for example, by proposing or introducing changes to terrorism legislation as it relates to younger age groups (e.g., Dodd, 2023; Renwick, 2018), or introducing new CVE programmes developed specifically for young terrorism offenders (e.g., Barracosa & March, 2022). However, a number of authors and organisations have cautioned against criminal justice responses to youth radicalisation owing to the ethical, legal, and practical challenges that can emerge when engaging children and adolescents through the criminal justice system (Ellis & Dalaine, 2022; Global Counterterrorism Forum [GCTF], 2015; Kessels, 2017; UNODC, 2021).

Because contact with the criminal justice system can be particularly distressing and stigmatising for young people, and can affect their life prospects (Global Counterterrorism Forum [GCTF], 2015; Kessels, 2017), efforts to counter youth radicalisation will benefit from considering and mitigating these potential harms. Engaging children and adolescents through preventive and diversionary interventions outside of the criminal justice system, for example, in community settings, is a potentially effective way of countering youth radicalisation in a way that can minimise these harms (Ellis & Dalaine, 2022; Global Counterterrorism Forum [GCTF], 2015; Kessels, 2017). These interventions are perceived to attract less stigma; may be considered more legitimate by potential participants; and may be better able to develop pro‐social relationships with clients (Kessels, 2017). However, the effectiveness of these interventions is poorly understood, and more research is needed to understand their effectiveness and implementation (Cherney, De Rooy, et al., 2022). This systematic review aims to address this key evidence gap.

### 1.2 The intervention

#### 1.2.1 Interventions for countering cognitive and behavioural radicalisation

Interventions working with children and adolescents span all three levels of CVE prevention (Elshimi, 2020). They include primary interventions that work to build resilience to radicalisation amongst young people more generally (Wolfowicz et al., 2022), as well as more targeted secondary and tertiary interventions. Secondary interventions work to interrupt or counter the radicalisation of young people who are considered at risk of engaging in violent extremism (Elshimi, 2020). The identification of this risk may be informed by an assessment indicating that an individual is vulnerable or susceptible to radicalisation (e.g., based on the presence of relevant risk factors), and/or is considered already on a radicalisation pathway. Tertiary interventions work with those already engaged in violent extremism (Cherney, De Rooy, et al., 2022) and work to promote deradicalisation and/or disengagement (Elshimi, 2020). Typically, those engaged in tertiary interventions actively support or have engaged in violent extremist activities (Vergani et al., 2020), including committing a relevant criminal offence, such as joining a proscribed terrorist group. These more targeted secondary and tertiary interventions include those specifically designed for youth cohorts (e.g., Barracosa & March, 2022; Hirschfield et al., 2012), as well as those that work with younger clients as part of broader caseloads (e.g., Home Office, 2023).

This review focuses solely on secondary and tertiary interventions working with at risk and radicalised young people, and excludes primary interventions. We do so for two reasons. First, due to their broader focus on building resilience, primary interventions are largely concerned with delivering outcomes whose relationship to cognitive and behavioural radicalisation is less direct (Marsden et al., 2017). Second, a comprehensive review of these interventions has already been completed by Wolfowicz et al. (2022), and directly informs this review's analytical approach.

Targeted secondary and tertiary interventions face scrutiny due to concerns about their effectiveness in countering radicalisation (Lewis, Marsden, Cherney, et al., 2024), and their impact on young people in particular (Cherney, De Rooy, et al., 2022). Key questions relate to whether and how existing secondary and tertiary interventions are able to meet the needs of children and adolescents (Cherney, De Rooy, et al., 2022) owing to those important distinctions between processes of youth and adult radicalisation discussed in Section 1.1. above.

The literature therefore recognises the importance of tailoring CVE approaches to younger cohorts, whether through developing interventions specifically for children and adolescents or engaging with younger clients in age‐appropriate ways in the context of broader interventions (Cherney, De Rooy, et al., 2022). However, existing review articles have concluded that the effectiveness of current interventions for countering youth radicalisation remains poorly understood (Cherney, De Rooy, et al., 2022; Wallner, 2021; White, 2021). This is particularly the case in the context of countering radicalisation amongst children and adolescents due to the conceptual ambiguity over terms such as ‘youth’, ‘childhood’ and ‘adolescence’, and associated challenges in identifying relevant interventions, and evidence of effectiveness. For example, because ‘youth’ is often understood as a broader stage of life that spans 15–24 years of age (Sawyer et al., 2018), even those CVE interventions that are tailored to ‘youth’ might need to be adapted further if they are to effectively work with children and younger adolescents.

Such evidence gaps are not restricted to interventions working with younger cohorts, instead reflecting broader weaknesses in the evidence base underpinning the field of CVE (Lewis, Marsden, Cherney, et al., 2024). However, specific concerns have been raised about CVE work with children and adolescents. These include the risk that, owing to their younger age, children and adolescents are more vulnerable to the types of negative psychological impacts that can result from interacting with different parts of the counterterrorism system (Hewitt et al., 2022). These concerns are particularly pronounced in the context of interventions working in and through the criminal justice system, which can be experienced as particularly distressing and stigmatising by younger people (Kessels, 2017). The co‐occurrence of mental health conditions in a number of child and adolescent cases of radicalisation raises further ethical challenges (Duits et al., 2022; Schulten, 2022) because criminal justice responses have the potential to exacerbate existing mental health issues (Sugie & Turney, 2017).

Such issues point to the important role played by CVE interventions that work with children and adolescents outside of the criminal justice system, for example, in community (e.g., Puigvert et al., 2020) or clinical (e.g., Rousseau et al., 2023) settings. The importance of delivering secondary interventions in non‐criminal justice contexts is relatively well‐established in the field of CVE owing to their focus on young people who have yet to become engaged in violent extremism, or committed a crime (Davies, 2023; Puigvert et al., 2020). Notwithstanding the potential for these interventions to produce ‘well‐intentioned harm’ through this work (Vaughn, 2019), these interventions have the potential to help divert young people away from violent extremism and the need for harder forms of intervention delivered through the criminal justice system.

Some tertiary interventions also operate outside of the criminal justice system, particularly those delivered by non‐governmental organisations (NGOs) (e.g., Christensen, 2015). However, it is common for tertiary interventions to be delivered in and through the criminal justice system – for example, in correctional or probation contexts. This is because they work with individuals who, by engaging in some form of violent extremist activity, are likely to have violated counterterrorism legislation (Cherney, De Rooy, et al., 2022). Some of these interventions also span secondary prevention by engaging with individuals convicted for non‐extremist crimes who have been identified as being at risk of radicalisation (e.g., Cherney & Belton, 2021).

The use of the criminal justice system in youth contexts raises ethical and legal issues. According to the principles of the United Nations Convention on the Rights of the Child, the arrest, conviction, and imprisonment of children must be a last resort, including in the context of terrorism offending (UNODC, 2021). The United Nations Office on Drugs and Crime (UNODC) also states that ‘[a]lternatives to prosecution and detention should normally and generally be favoured’ (p. 50) when working with children (i.e., those aged under 18 years of age) in the context of countering terrorism.

The severity of some recent cases of adolescent radicalisation (Wallner, 2021) highlights that there may be no feasible alternative to detention in some instances; because of the threat posed by the individual, or when the failure to prosecute individuals for particular offences undermines the ethical and legal obligations governments have towards victims (UNODC, 2021). However, the intent, capability, and capacity for violence can vary markedly amongst young people convicted for different types of online and offline terrorism offences (Rose & Vale, 2023). This suggests that some of these individuals may not pose a significant threat of terrorist violence and could feasibly be managed outside of the criminal justice system. As well as being less distressing for the individual, and better able to motivate them to pursue positive change (Kessels, 2017), there is evidence from the broader field of violence prevention that diverting first‐time or lower‐order youth offenders outside of the criminal justice system can be effective in countering violence (Gaffney et al., 2021).

There appear to be practical and ethical benefits in seeking to prevent and counter childhood and adolescent radicalisation through secondary and tertiary interventions delivered outside of the criminal justice system. However, the reviews cited above highlight that the evidence base underpinning such interventions remains underdeveloped. No existing reviews have yet comprehensively synthesised evidence relating to how these interventions are being implemented in practice. The growing numbers of young people who are now being engaged through secondary and tertiary interventions means that it has become increasingly important to understand whether and how these interventions work to deliver positive outcomes relevant to countering radicalisation. This review aims to address this gap by examining the effectiveness and implementation of interventions working to counter the radicalisation of those aged 19 years or under outside the criminal justice system.

#### 1.2.2 Interventions beyond the boundaries of the criminal justice system

Determining whether interventions operate outside of the criminal justice system can be challenging because the boundaries of this system are hard to delineate (Levin, 2023). A key challenge relates to the increasing collaboration between criminal justice agencies such as the police, and professionals working outside of the criminal justice system (Levin, 2023). This challenge is particularly acute in the context of CVE due to the pervasiveness of multi‐agency interventions that include criminal justice agencies as a partner, or that are led by criminal justice agencies (Mazerolle et al., 2021).

Previous Campbell Collaboration reviews have defined criminal justice CVE interventions on the basis that at least one criminal justice agency, such as the police, courts, or corrections is involved (e.g., Sydes et al., 2022). Whilst this definition is useful, criminal justice agencies are often important partners in interventions that work with young people before they have committed any crime (e.g., Thompson & Leroux, 2022), as well as those offered as alternatives to formal processing through the criminal justice system (e.g., Wilson et al., 2018). Adopting a similar definition for this review would therefore mean excluding a range of interventions that work to prevent children and adolescents from ever requiring a formal intervention through the criminal justice system (i.e., at the arrest, prosecution, or imprisonment stage), or which aim to divert them away from the criminal justice system altogether. This emphasis on diversion and prevention is a defining feature of a non‐criminal justice intervention.

In order to capture the broadest possible range of relevant interventions, we therefore restrict our definition of criminal justice interventions to those that are delivered *by* criminal justice agencies (e.g., the police, correctional, or probation staff) *through* the criminal justice system to individuals prosecuted and convicted of relevant offences (e.g., in correctional, custodial, or post‐release contexts). In the broadest sense, the objectives of these interventions are comparable to interventions operating outside of the criminal justice system in that both aim to prevent and counter future or ongoing (re)engagement in violent extremism. As noted above, both criminal and non‐criminal justice interventions can also span secondary and tertiary prevention, and in turn seek to tackle cognitive and/or behavioural radicalisation, although the emphasis may vary. By definition, criminal justice interventions might be expected to more commonly work with clients who exhibit more severe forms of (behavioural) radicalisation, particularly those working in post‐conviction settings. In contrast, interventions outside of the criminal justice system might be expected to more commonly work with those at an earlier stage of (cognitive) radicalisation, and may even refer individuals at later stages of radicalisation to criminal justice agencies (HM Government, 2023). However, this distinction is not absolute: some criminal justice interventions work with individuals who are at an earlier stage of radicalisation (e.g., Cherney & Belton, 2021), whilst some non‐criminal justice interventions work with clients who have been behaviourally radicalised, including those who have joined violent groups (e.g., Christensen, 2015).

Instead, the fundamental distinction between the two types of intervention is that in contrast to interventions that operate through the criminal justice system as a consequence of arrest, prosecution, or imprisonment, interventions operating outside of the criminal justice system work to prevent or divert individuals away from arrest, prosecution, and imprisonment. This review therefore defines a non‐criminal justice intervention as any intervention that works to prevent young people from entering, or divert them away from, the criminal justice system, including those that involve some form of criminal justice agency participation. Examples include:

- Interventions working with at risk or radicalised young people that operate *completely independently* of the criminal justice system, such as community‐led programmes delivered by NGOs.
- Interventions engaging with youth at risk of radicalisation *before* they have committed or have been investigated for a violent extremist or terrorism offence, including those that partner with criminal justice agencies such as the police.
- Interventions designed to divert individuals who have been investigated or arrested for an offence away from the criminal justice system. This type of diversionary activity might occur at the point of arrest, before charges are made, or at the point of sentencing (Gaffney et al., 2021).

### 1.3 How the intervention might work

With some exceptions, the theories of change or programme logics underpinning secondary and tertiary CVE interventions are rarely made explicit (Lewis, Marsden, Cherney, et al., 2024). However, these interventions commonly work by identifying individuals who are at risk of engaging in (secondary interventions), or already engaged in (tertiary interventions) violent extremism, and targeting relevant risk factors and/or protective factors to prevent and counter cognitive and behavioural radicalisation (Wolfowicz et al., 2021). This includes more risk‐oriented approaches that are primarily oriented towards risk management and reduction, and strengths‐based approaches which focus on building skills and strengths believed to contribute to longer term resilience (Marsden, 2017).

In the context of secondary interventions, at risk young people may be identified through a variety of different mechanisms, including through referrals from family members, friends, or front‐line staff working in different settings (Lewis, Marsden, Cherney, et al., 2024). The same mechanisms may also be used within tertiary interventions (Costa et al., 2021), although eligible individuals are often primarily identified when they have committed a relevant terrorism or violent extremist offence (Barracosa & March, 2022). In contrast to secondary interventions which largely work with clients before they have committed a crime, tertiary interventions are often delivered through the criminal justice system. However, young offenders who come into contact with the criminal justice system may be diverted to interventions operating outside the criminal justice system as an alternative to arrest, prosecution, or imprisonment (Gaffney et al., 2021).

Interventions operating outside of the criminal justice system vary according to the type and focus of support they offer to at risk or radicalised young people. Some interventions may tailor the support offered to individual clients (Lewis, Marsden, Cherney, et al., 2024), whilst others may offer more fixed packages of support, for example, using a set curriculum (e.g., Feddes et al., 2015; Webber et al., 2018). The content of an intervention can also vary, and might include a combination of educational, psychological, ideological, theological, or vocational support (Cherney & Belton, 2021), delivered on a one‐to‐one basis (e.g., Christensen, 2015), through group‐based work (e.g., Feddes et al., 2015), or some mixture of the two. A number of interventions also work with and through familial, community, or social networks to prevent and counter the radicalisation of a young person (Haugstvedt, 2022). Regardless of the type and focus of support, these differing interventions are underpinned by the assumption that youth radicalisation can be tackled through activities that aim to reduce risk factors, and/or build protective factors for radicalisation.

A key consideration when working with children and adolescents is the importance of delivering interventions in ways that are age‐appropriate (Barracosa & March, 2022). This means tailoring the content and aims of interventions so that they take account of the specific needs of younger cohorts, and the developmental differences that exist between children and adolescents of different ages (Cherney, De Rooy, et al., 2022). It also means being sensitive to the particular forms of psychological and developmental harms that exposure to violent extremist content and individuals, including extremist family members (Rousseau et al., 2024), can produce for children and adolescents (Koehler, 2020), as well as the risk of stigma and distress that can result from efforts to intervene in youth radicalisation (Global Counterterrorism Forum [GCTF], 2015; Kessels, 2017). Such considerations are not restricted only to interventions working with children and adolescence, but are likely to be particularly acute when supporting younger cohorts.

### 1.4 Why it is important to do this review

This systematic review aims to support practitioners and policymakers working to tackle childhood and adolescent radicalisation by addressing key evidence gaps relating to interventions delivered outside of the criminal justice system. By synthesising evidence relating to effectiveness and implementation, this review aims to support the design and delivery of interventions working in this context by (a) identifying interventions proven to be effective, and that could be used to inform other, comparable programmes; and (b) identifying implementation factors and moderators that facilitate (or inhibit) the delivery of relevant interventions, and that should be considered when designing and implementing comparable programmes. Despite growing recognition of the importance of these interventions, no ongoing or completed systematic review has focused on this type of intervention specifically.

Several reviews have specifically focused on youth interventions in this context, most notably Cherney, De Rooy, et al. (2022), Wallner (2021), and White (2021). These reviews contain important insights that are highly relevant. However, whilst robust, they do not fully conform to the methodological approach for identifying and assessing studies used in a Campbell Collaboration review and include a broader range of studies than would meet the inclusion criteria for this review. They also have a different focus to this review: Cherney, De Rooy, et al. (2022) include insights from primary interventions and interventions operating in criminal justice contexts, whereas Wallner (2021) and White (2021) focus solely on secondary interventions. And, whilst these reviews were published relatively recently, the fact that research and practice is now increasingly concerned about youth radicalisation as noted above also means that an updated review is needed at this time, as it is likely that a number of important studies have been published in the years since the searches for these reviews were conducted.

Important insights are also captured in reviews that are not specifically focused on youth interventions. Examples include reviews conducted by members of this research team (Lewis, Marsden, Cherney, et al., 2024); and by Hassan et al. (Hassan, Brouillette‐Alarie, Ousman, Kilinc, et al., 2021a; Hassan, Brouillette‐Alarie, Ousman, Savard, et al., 2021b) who included search terms relating to youth cohorts in their search strategy, but did not restrict their inclusion criteria to interventions working with youth or that are delivered outside of the criminal justice system. Whilst again useful, the challenges associated with working with children and adolescents in this context mean that it cannot be assumed that insights drawn from interventions working with older cohorts can be extrapolated to efforts to counter childhood and adolescent radicalisation (Cherney, De Rooy, et al., 2022).

Conceptual ambiguity around the definition of ‘youth’ means that a more targeted review is necessary to provide insights into the issues relevant to those aged 19 years or under. A more comprehensive review is also needed to ensure that all relevant studies are captured, particularly those that are not always indexed in the most commonly searched databases. Moreover, existing reviews are not restricted to interventions operating outside of the criminal justice system, and do not examine whether this particular type of intervention is effective, nor whether and how this age‐related delivery context might facilitate or inhibit how interventions work with clients. This limits their ability to speak to our research objectives (see below).

The above challenges link to broader weaknesses in the evidence base. As well as a limited understanding of whether interventions are effective, scant attention has been paid to whether interventions are implemented in ways which are likely to support positive outcomes. Previous Campbell reviews (Lewis, Marsden, Cherney, et al., 2024; Mazerolle et al., 2021) have examined the implementation of relevant interventions as part of broader reviews of case management and multi‐agency programmes. However, owing to their broader focus, neither review focuses specifically on issues relevant for children and adolescents, which limits their ability to speak to our objectives. Those previous reviews of youth interventions discussed above have provided important insights relating to implementation, but this analysis is more limited than contained within the Campbell reviews by Lewis, Marsden, Cherney, et al. (2024) and Mazerolle et al. (2021). The synthesis of evidence on implementation will therefore be a particularly important contribution.

This twin focus on effectiveness and implementation is informed by a previous review conducted by members of this research team on case management interventions (Lewis, Marsden, Cherney, et al., 2024) which was unable to find any eligible evaluations of effectiveness. This was unsurprising given the well‐established practical and ethical challenges undermining efforts to use experimental and quasi‐experimental research when evaluating secondary and tertiary CVE interventions (Lewis, Marsden, Cherney, et al., 2024). It is likely that the current review will face similar challenges in trying to identify evidence of effectiveness which makes it particularly important to capture data on implementation.

Although research on implementation cannot be used to examine *whether* interventions work, it can be used to examine *how* they work; that is, whether they are being delivered in ways that are likely to produce positive effects on youth radicalisation. Through this twin focus, we aim to develop understanding of how the interventions examined in this review are being implemented, and those factors that inhibit or facilitate their implementation. The current review therefore aims to build on a modest but growing body of evidence that focuses more explicitly on the processes and mechanisms that support intervention design, delivery, and evaluation (Lewis, Marsden, Cherney, et al., 2024) to complement the emphasis on questions of impact and effectiveness.

## 2 OBJECTIVES

This review aims to answer the three research questions and corresponding objectives listed in Table 1.

**Table 1:** Research questions and objectives.

RQ	Question	Objective
1	Are secondary and tertiary interventions delivered outside of the criminal justice system effective at countering the cognitive and behavioural radicalisation of children and adolescents?	Synthesise evidence relating to relevant primary and secondary outcomes of effectiveness.
2	Are secondary and tertiary interventions delivered outside of the criminal justice system being implemented as intended?	Synthesise evidence that captures how interventions are implemented, considering whether they are implemented as expected or in ways that align with their underlying logic.
3	What factors influence how interventions working with children and adolescents outside of the criminal justice system are implemented?	Identify those implementation factors (facilitators and barriers) and moderators that impact how interventions are delivered.

## 3 METHODS

### 3.1 Criteria for considering studies for this review

#### 3.1.1 Types of studies

##### Evaluating effectiveness (Objective 1)

Only quantitative studies using experimental or ‘stronger’ quasi‐experimental designs as defined in previous Campbell reviews will be eligible for inclusion when evaluating effectiveness (Lewis et al., 2023; Mazerolle et al., 2021). We are guided by previous Campbell Collaboration reviews when defining ‘stronger’ designs, in particular Mazerolle et al. (2021). However, this review will only include designs that allow for a comparison of a treatment group (i.e., intervention group) to a reasonable comparison group as defined below (e.g., matched control group, control group with face validity). Given the noted absence of evaluations using experimental designs in this field, the comparison group will be assessed on a case‐by‐case basis, and the type of comparison will be coded and analysed as a moderator. Adapting the list used by Mazerolle et al. (2021), eligible designs will include:

- Randomised controlled trials (RCTs).
- Cross‐over designs (randomised and non‐randomised).
- Propensity or statistically matched control group designs (with or without baseline).
- Unmatched control group designs without baseline where control group has face validity.
- Unmatched control group designs with pre‐post intervention measures allowing for difference‐in‐difference analysis.
- Short interrupted time‐series designs with control group (less than 25 pre‐and post‐intervention observations).
- Long interrupted time‐series designs with or without a control group (over 25 pre‐and post‐intervention observations).

Due to known challenges in using control groups when evaluating secondary and tertiary CVE interventions (Lewis et al., 2020), no restrictions will be set on comparator conditions used in quasi‐experimental or experimental designs. Eligible conditions will include waitlist; treatment‐as‐usual; no treatment; and alternative treatment.

##### Evaluating implementation (Objectives 2 and 3)

A broader range of research designs will be eligible for inclusion in the analysis of implementation. In addition to the experimental and stronger quasi‐experimental research designs identified above, this analysis will also include data from quantitative studies using quasi‐experimental and non‐experimental designs, and qualitative data drawn from qualitative and mixed‐methods studies. Data from studies using these research designs will be crucial given the continued challenges facing efforts to use stronger quasi‐experimental and experimental designs discussed above.

The inclusion of qualitative and non‐experimental research designs is informed by our previous analysis of implementation (Lewis, Marsden, Cherney, et al., 2024), and by authors such as Higginson et al. (2015), who have argued for including ‘the broadest range of evidence that assesses the reasons for implementation success or failure' (p. 22). Data drawn from studies using these designs – particularly qualitative studies – hold valuable insights into those factors influencing how interventions are implemented and will help inform a more holistic analysis of intervention processes and outcomes.

Studies included in the analysis of effectiveness will also be included in the analysis of implementation when they report on relevant implementation factors and moderators of implementation as defined below. An overall assessment of the strength of evidence relating to each implementation factor and moderator will be included owing to the fact that this analysis will synthesise evidence from experimental, quasi‐experimental, and non‐experimental quantitative studies, and qualitative studies. This assessment will summarise the types of research design included in the analysis of each factor/moderator, and their quality/risk of bias (see Section 3.3.4).

#### 3.1.2 Types of participants

This review focuses specifically on childhood and adolescence as distinct stages of life, as opposed to the broader concept of youth which, as noted above, is often defined as extending up until 24 years of age.^1^ This narrower focus reflects the interest in children and adolescents specified in the original brief for the project.^2^ More substantively, this decision recognises that childhood and adolescence are distinctive stages of development during which the needs of individuals will differ to those of a young adult (Barracosa & March, 2022). Whilst there is no single definition of childhood and adolescence, common definitions of both stages of life often overlap. For example, the UN Convention on the Rights of the Child defines children as those under 18 (UNODC, 2021), whilst the WHO defines adolescence as the period from 10 to 19 years of age (WHO, undated). Following these definitions, we focus on interventions working with those aged 19 years or under, although we recognise some authors argue for extending the definition of adolescence further (Sawyer et al., 2018).

Studies reporting on secondary or tertiary interventions that work to prevent or counter the radicalisation of at risk (secondary) or radicalised (tertiary) individuals aged 19 years and under will be eligible. This includes interventions working directly with these cohorts, and/or with family members and/or broader social networks of at risk or radicalised children and adolescents seeking to prevent further radicalisation. To be eligible for inclusion, interventions must therefore work with at least one of the below:

- Clients explicitly described as being at risk of radicalisation or at an early stage of radicalisation and/or who have been assessed as such according to a specified set of criteria, for example, using a particular risk assessment tool, or based on their association with individuals who have become radicalised.
- Clients who have been cognitively or behaviourally radicalised, as evidenced by, for example, a conviction for a relevant extremist offence.
- Family members or other contacts of at risk or radicalised young people aged 19 years or under, when the express focus of this engagement is to prevent or counter the radicalisation of the young person in question.

There will be no additional socio‐demographic exclusion criteria.

Eligible interventions will include those working exclusively with clients aged 19 years or under, and those that work with this cohort as part of broader caseloads. Interventions working with broader caseloads may include those that work with children, adolescents, and adults; and those that are specifically designed for ‘youth’ cohorts (or related constructs), but which use a broader definition of youth as defined above or which do not make the age range of clients explicit (in which case, we will attempt to obtain this information from study authors). We will use objective‐specific criteria to determine whether studies reporting on interventions working with broader caseloads, or non‐defined youth caseloads are eligible for inclusion in the analysis:

- Effectiveness: Outcome data specific to youth (or related) constructs must be presented in the study or available from the study author(s). Wherever possible, we will isolate (or request) data specific to clients aged 19 years or under. When this data is not available, outcome data specific to ‘youth’ (or related constructs) presented in the study or available from the author will still be included in the meta‐analysis (e.g., when an intervention uses a broader definition of youth, or a study of a youth intervention does not specify the age range of clients). However, we will examine the effects of including these broader samples in the meta‐analysis through the use of sensitivity analysis which is discussed below.

- Implementation: Studies will be eligible for inclusion when they meet at least one of two conditions: (1) The caseload of the examined intervention must include 50% or more participants aged 19 years or under; (2) Studies must report on findings specific to working with youth clients (however defined).

#### 3.1.3 Types of interventions

This review focuses on secondary and tertiary interventions working to counter the radicalisation of young people aged 19 years or under. To be included, studies must therefore report on a secondary or tertiary intervention working to counter cognitive and/or behavioural radicalisation as defined above. This includes standalone interventions that focus solely on countering violent extremism, or interventions that aim to counter violent extremism and other harms (Thompson & Leroux, 2022). As noted above, this review excludes primary interventions due to their broader focus.

A diverse range of secondary and tertiary interventions work with children and adolescents outside of the criminal justice system. Examples of eligible interventions might include, but are not limited to, multi‐agency case management interventions that tailor support to the needs of each individual client (Lewis, Marsden, Cherney, et al., 2024); mentoring programmes (Christensen, 2015); and dialogical approaches that aim to challenge violent extremist beliefs by bringing at risk and radicalised young people into dialogue with others from outside their own extremist milieu (Hussain et al., 2019; Skiple, 2020). Some interventions might adopt less direct methods, for example, by supporting parents or peers to intervene in a young person's radicalisation (Haugstvedt, 2022). A variety of practitioners may be involved in delivering these interventions in different settings, including community‐based organisations, mental healthcare professionals, social workers, mentors, and dedicated CVE practitioners (Lewis, Marsden, Cherney, et al., 2024).

Interventions working to counter radicalisation relating to any form of violent extremism will be eligible. This includes those working to counter domestic forms of violent extremism as captured in categories used by DHS and the Federal Bureau of Investigation (FBI) (i.e., Racially or Ethnically Motivated Violent Extremism; Anti‐Government or Anti‐Authority Violent Extremism; Animal Rights/Environmental Violent Extremism: Abortion‐Related Violent Extremism; All Other Domestic Terrorism Threats)^3^; as well as international forms of violent extremism, such as those associated with Foreign Terrorist Organisations as designated by the United States.^4^

When assessing if an intervention works to counter radicalisation as defined above, we will be guided by the definitions used by the US government, as well as common definitions used in the literature, including by Vergani et al. (2020). Interventions will be assessed by examining their objectives and aims as outlined in a study (if listed), and the extent to which it explicitly aims to counter cognitive or behavioural radicalisation as defined above. Where intervention aims and objectives are not explicit, eligibility will be determined based on assessing whether the intervention works with radicalised or at‐risk youth as defined above, and whether the outcomes used to evaluate the intervention relate to cognitive or behavioural radicalisation.

There is a lack of agreement in the literature as to how best to identify individuals at risk of radicalisation, and the risk factors that might be used for this purpose (Corner & Taylor, 2022). Rather than use specific indicators of risk, we will therefore rely on the descriptions of interventions, and intervention clients, when determining whether an intervention works with young people who are at risk of radicalisation and should therefore be classed as a secondary intervention. To be defined as such, interventions must be described as a secondary intervention and/or as working with those at risk of, or at an early stage of, radicalisation as defined in Section 3.1.2 above.

The line between secondary and tertiary prevention is somewhat blurred, as the extent to which clients are considered to be at risk of radicalisation, or already radicalised, will depend on how the term radicalisation is defined. However, when defining tertiary interventions, we will use a similar approach as for secondary interventions. To be defined as such, interventions must specifically be described as tertiary interventions or as working with individuals who have been cognitively or behaviourally radicalised, as defined in Section 3.1.2 above. One potential area of complexity here relates to competing conceptualisations of interventions that work with current or former members of violent extremist groups engaged in larger‐scale militancy in conflict zones. Interventions such as Operation Safe Corridor in Nigeria and the Serendi rehabilitation centre in Somalia work with former members of foreign terrorist organisations as designated by the United States (Boko Haram and Al‐Shabaab, respectively) but are often defined as Disarmament, Demobilisation and Reintegration (DDR) programmes, as opposed to CVE interventions (Khalil et al., 2019; Richards, 2017). Notwithstanding the important conceptual distinctions between CVE and DDR, such interventions would meet our definition of tertiary interventions owing to the fact that they have specifically been described as working with ‘violent extremists’ (e.g., Khalil et al., 2019; USAID, 2021), indicating cognitive and/or behavioural radicalisation. Interventions delivered in the context of larger‐scale militancy (i.e., DDR interventions) will therefore be included in the review (when meeting our other criteria) when it can be determined that they work with relevant clients as defined above.

#### 3.1.4 Types of outcome measures

##### Outcomes relating to effectiveness (Objective 1)

There is no single metric against which to assess the effectiveness of counter‐radicalisation interventions (Cherney et al., 2018). Therefore, this review draws on previous systematic reviews to identify relevant primary and secondary outcomes for assessing the effectiveness of an intervention at countering youth radicalisation. Studies reporting on primary and/or secondary outcomes – including those that report on secondary outcomes only – will be eligible for inclusion.

Primary outcomes are defined according to a typology of different radicalisation outcomes identified in Wolfowicz et al.'s (2021) Campbell Collaboration systematic review of risk and protective factors for cognitive and behavioural radicalisation. Based on a definition of radicalisation as a process resulting in ‘the development of attitudes supportive of the use of violence in the name of a cause’ and/or ‘the carrying out of such violence’ (Wolfowicz et al., 2021, p. 2), this original typology distinguished between radical attitudes, radical intentions, and radical behaviours as shown below:

- Radical attitudes: Justification/support for radical behaviours carried out in the name of a cause.
- Radical intentions: Willingness/intentions towards engagement in radical behaviours in the name of a cause.
- Radical behaviours: Actual involvement in violent radical behaviours in the name of a cause, including terrorism (Wolfowicz et al., 2021, p. 12).

We adopt this same typology of attitudes/intentions/behaviours. However, in line with our own definition of radicalisation presented earlier, we use the language of violent extremism as opposed to radicalism. We will therefore include studies that capture the impact of an intervention on the following primary outcomes:

- Violent extremist attitudes (e.g., the impact of an intervention on clients' support/sympathy for a violent extremist or terrorist cause, movement, or organisation).
- Violent extremist intentions (e.g., the impact of an intervention on clients' willingness or stated intention engage in violent extremist action, such as joining a violent extremist or proscribed terrorist organisation).
- Violent extremist behaviours (e.g., the impact of an intervention at preventing engagement in violent extremism, as evidenced by, e.g., a client's disengagement and desistence from an extremist or terrorist group).

Following Wolfowicz et al. (2021), all relevant measures will be categorised into one or more of the above categories of primary outcomes for the purposes of analysis. Outcomes relating to violent attitudes/intentions/behaviours that are not associated with a violent extremist or terrorist ideology as defined earlier will not be eligible for inclusion, and so studies that only report on such outcomes will be excluded.

Secondary outcomes relate to those dynamic risk and protective factors that are potentially amenable to treatment that have been empirically linked to cognitive and/or behavioural radicalisation in the same review by Wolfowicz et al. (2021). That review identified different domains of risk and protective factors as shown in Table 2 below.

**Table 2:** Example secondary outcomes (based on Wolfowicz et al., 2021).

Domain	Examples
Socio‐demographic/background	*Risk factors* Unemployment, alcohol or substance abuse, relationship problems, etc. *Protective factors* Socio‐economic status, education, etc.
Attitudinal^a^	*Risk factors* Perceived in‐group superiority, perceived discrimination, perceived injustice, etc. *Protective factors* Law abidance, belief in legitimacy of law, trust in institutions, trust in others, etc.
Psychological/personality	*Risk factors* Mental health issues, anger, negative affect, authoritarianism, etc. *Protective factors* Life satisfaction, higher self‐esteem, etc.
Experiential	*Risk factors* Family violence, personal strain, etc. *Protective factors* High perceptions of procedural justice.
Criminogenic	*Risk factors* Deviant/radical peers, low self‐control, thrill seeking, etc. *Protective factors* Parental involvement, school bonding, outgroup friends, etc.

^a^ This category is distinct to the primary outcome of ‘attitudes’. In this context, attitudinal secondary outcomes relate to attitudes that are believed to indicate an increased risk of cognitive/behavioural radicalisation, whereas the primary outcome ‘attitudes’ relates to attitudes indicative of radicalisation.

Studies reporting on a relevant intervention's impact on any of the risk and protective factors identified in Wolfowicz et al.'s (2021) original review will be eligible for inclusion in the review, provided that these outcomes are specifically identified as being relevant to countering cognitive or behavioural radicalisation. Studies that only report on similar outcomes without reference to cognitive or behavioural radicalisation (e.g., studies reporting on other types of violence prevention programmes) will not be eligible.

Data relating to these outcomes may be captured using a variety of data collection tools and approaches. Examples might include primary research data (e.g., surveys, interviews); secondary analysis of programme documentation (e.g., risk assessment or case note data); and evidence relating to recidivism (e.g., arrest data). No restrictions will be placed on the method, tool, or approach used to capture outcomes, and outcome data collected from practitioners, stakeholders, at risk and radicalised young people, and family members and other contacts will be eligible for inclusion.

##### Outcomes relating to implementation (Objectives 2 and 3)

No restrictions on outcomes will be set when examining implementation, in line with previous Campbell Collaboration reviews analysing the implementation of CVE interventions (Lewis, Marsden, Cherney, et al., 2024; Mazerolle et al., 2021). However, interventions must specifically work to deliver relevant primary or secondary outcomes as defined above to be included in this analysis of implementation. The focus of Objective 2 will be data relating to how interventions are implemented in practice, and whether they are being implemented as intended or expected. This permits an assessment of whether an intervention is being implemented in ways that are likely to produce positive outcomes, even when robust evidence of effectiveness is lacking.

Effective implementation is likely to rest on an intervention being underpinned by sound assumptions about how to address radicalisation, and a clear logic through which these assumptions are translated into practice (Lewis, Marsden, Cherney, et al., 2024). Ideally, this would be articulated in a clearly defined theory of change that links programme aims to activities and intended outcomes. This can be hard to determine as theories of change are rarely articulated explicitly, and the assumptions underpinning interventions are largely implicit. Nevertheless, our previous review was able to assess the validity of explicit or implicit logics underpinning CVE interventions; the implementation challenges that might arise when these logics are flawed; and/or whether and how interventions were implemented in ways that were consistent with these logics, and therefore likely to produce positive outcomes (Lewis, Marsden, Cherney, et al., 2024).

Objective 3 focuses on studies reporting on implementation factors and moderators found to influence how interventions are implemented. Implementation factors and moderators are defined in line with our earlier review (Lewis, Marsden, Cherney, et al., 2024). Implementation factors refer to ‘actions or actors necessary to successfully install and maintain an intervention’ (Thornton et al., 2019, p. 267 in Cherney et al., 2020, p. 16), categorised into factors that act as facilitators or barriers to implementation, and moderators to the ‘contextual conditions’ or ‘features of the people or places that are the target for intervention’ (Thornton et al., 2019; p. 267 in Cherney et al., 2020, p. 15).

Key implementation factors identified in a previous Campbell Collaboration review that may be relevant to interventions for children and adolescents include effective multi‐agency working, resourcing, the ability to tailor interventions to clients and contexts, practitioner characteristics and approaches, and expertise and experience (Lewis, Marsden, Cherney, et al., 2024). Key moderators are likely to include the delivery and local context in which an intervention operates (Lewis, Marsden, Cherney, et al., 2024). Data relating to the moderating effect of delivery contexts will be pertinent given our interest in exploring whether the perceived benefits of working with young people outside of the criminal justice system are reflected in practice. The review will also examine whether specific tools or methods – such as risk assessment tools or case conferences – support practitioners in delivering interventions in ways likely to produce positive outcomes, for example, enabling them to assess and meet client needs more effectively (Lewis, Marsden, Cherney, et al., 2024).

##### Duration of follow‐up

No restrictions will be set on the length of follow‐up period. In the event of any differences in the follow‐up period used in included studies, comparable follow‐up periods will be grouped into one of the following categories for the purposes of analysis: 0 to ≤3 months post‐intervention, >3 ≤6 months post‐intervention, >6 ≤12 months post‐intervention and >12 months post‐intervention (although, these categories may be adjusted based on actual periods to make for meaningful comparisons). We will then include each follow‐up period as a predictor in meta‐regression.

#### 3.1.5 Types of settings

Only interventions delivered outside of the criminal justice system will be eligible for inclusion. As noted above, criminal justice interventions are defined as those interventions that are delivered in correctional, custodial, or post‐release contexts. Any intervention delivered outside of these contexts – for example, in community, clinical, educational, or social work settings – will be included provided that they meet one of the below criteria:

1. Work with individuals before they formally come into contact with the criminal justice system (i.e., before any criminal investigation or arrest).
2. Provided as an alternative to arrest, charge, or imprisonment to individuals who are/have previously been subject to a criminal investigation or arrest.
3. Work with individuals who have previously been or are subject to a criminal investigation, arrest, prosecution, and/or sentence but which operate independently from criminal justice agencies, or the criminal justice system.

Interventions covered by points 1 and 2 may see some involvement by criminal justice agencies, for example, where the police, or multi‐agency panels involving criminal justice agencies, refer individuals into programmes (e.g., Thompson & Leroux, 2022). Because the role criminal justice agencies play within these processes can vary, we will only exclude interventions when a criminal justice agency plays a role in working directly with clients. No other restrictions will be placed on delivery agents, provided that interventions are delivered outside of, or aim to divert individuals from deeper engagement in, the criminal justice system (Wilson et al., 2018).

#### 3.1.6 Publication types

Eligible peer‐reviewed and non‐peer‐reviewed/grey literature studies will be included. Protocols and trial registries for eligible ongoing studies for which no published outputs can be identified will be included in a separate ‘References to ongoing studies’. However, should they link to a finalised study, such references will be classed as a dependent report of that study.

#### 3.1.7 Countries

There are no geographical exclusion criteria.

#### 3.1.8 Languages

Research published in English will be included.

#### 3.1.9 Date of publication

Studies published after January 2000 will be included. Whilst some relevant interventions have a longer history, the policy framework of countering violent extremism only really emerged at the start of the 21st century (Neumann, 2013).

### 3.2 Search methods for identification of studies

Studies will be identified using an updated version of the search strategy used in our previous review of case management interventions (Lewis, Marsden, Cherney, et al., 2024):

1. Targeted systematic searches of academic platforms (Table 4).
2. Hand searches of key journals (Table 5); grey literature sources related to countering violent extremism (Table 6); and clinical trial repositories (Table 7).
3. Bibliographic searches of existing, relevant synthesis papers (Table 8).
4. Consulting with relevant experts.
5. Forward and backward citation searching of studies identified at Stages 1–4.

Each of these steps is examined in detail below.

#### 3.2.1 Electronic searches

An information specialist (DS) developed a comprehensive core search strategy comprised of subject headings and keyword search terms in Ovid PsycINFO in consultation with the primary authors (JL/SM) via an iterative process through which the search terms from an earlier review (Lewis, Marsden, Cherney, et al., 2024) were adapted based on reviewing examples of studies eligible for inclusion in this review; reorienting the search terms to focus more explicitly on interventions for young people aged 19 years or under; and a series of stages of piloting. We present the full strategy for PsycINFO in Table 3. The information specialist will model strategies for the remaining databases (Table 4) on the strategy developed for PsycINFO, using appropriate syntax for each database and platform. Search fields for all databases will include, as a minimum, title, abstract, author supplied keywords, and controlled vocabulary indexing terms.

**Table 3:** Core search strategy.

Database: APA PsycINFO
Search Strategy: ‐‐‐‐‐‐‐‐‐‐‐‐‐‐‐‐‐‐‐‐‐‐‐‐‐‐‐‐‐‐‐‐‐‐‐‐‐‐‐‐‐‐‐‐‐‐‐‐‐‐‐‐‐‐‐‐‐‐‐‐‐‐‐‐‐‐‐‐‐‐‐‐‐‐‐‐‐‐‐‐
1 (neonatal birth 1 mo or infancy 2 23 mo or preschool age 2 5 yrs or school age 6 12 yrs or adolescence 13 17 yrs or young adulthood 18 29 yrs).ag.
2 (adolesc* or boy* or child* or daughter or daughters or girl* or juvenile* or minor or minors or preadolesc* or prepubescen* or puberty or pubescen* or son or sons or teen* or ‘young adult*’ or young* or ‘young people’ or youth*).mp.
3 (college* or ‘elementary school*’ or ‘high school*’ or ‘junior high’ or ‘middle school*’ or ‘primary school*’ or pupil* or ‘secondary school*’ or ‘school‐age*’ or ‘school age*’ or student* or universit*).mp.
4 or/1‐3
5 extremism/
6 exp terrorism/
7 radical movements/
8 (extreme‐left or extreme‐right or ‘extreme violen*’ or extremism or extremist or extremists).mp.
9 terroris*.mp.
10 (anarch* or bioterror* or far‐left or far‐right or incel or incels or indoctrinat* or islamis* or jihadi* or militant* or militia*).mp.
11 (nationalis* or neo‐nazi* or radicali* or radical or radical* or salafi* or ‘violent ideolog*’ or ‘white supremacis*’).mp.
12 (radical* adj3 (belief* or group* or ideolog* or left* or movement* or organisation* or organization* or right* or theolog*)).mp.
13 or/5‐12
14 risk management/
15 school based intervention/
16 interven*.mp.
17 ((alternative* or approach* or ‘at risk’ or campaign* or communit* or dialog* or divert* or diversion?) adj10 (counter* or deradical* or de‐radical* or desist* or disengag* or exit* or prevent* or recidivis* or reduc* or re‐enter* or reentry or re‐entry or rehab* or reintegrat* or re‐integrat*)).mp.
18 ((engage* or engaging or factor* or guidance or implement* or initiative* or interact*) adj10 (counter* or deradical* or de‐radical* or desist* or disengag* or exit* or prevent* or recidivis* or reduc* or re‐enter* or reentry or re‐entry or rehab* or reintegrat* or re‐integrat*)).mp.
19 ((method* or model* or program* or policy or policies or practice* or project* or scheme* or strateg* or treat*) adj10 (counter* or deradical* or de‐radical* or desist* or disengag* or exit* or prevent* or recidivis* or reduc* or re‐enter* or reentry or re‐entry or rehab* or reintegrat* or re‐integrat* or ‘risk manage*’)).mp.
20 ((alternative* or counter* or mitigat* or prevent* or reduc* or rehab* or reintegrat* or re‐integrat* or ‘risk manage*’ or stop*) adj5 (extremis* or radicali* or terrori*)).mp.
21 or/14‐20
22 4 and 13 and 21
23 animal.po.
24 exp animals/or animal models/or exp animal research/or exp primates/(397466)
25 or/23‐24
26 human.po.
27 25 not 26
28 ((afrik* or alban* or arab* or bulgar* or catalan or chinese or croat* or cze* or danish or dut* or estonian or fars* or finn* or french or georgian or ger* or gre* or hebr* or hindi or hungarian or ital* or japan* or kor* or latv* or lith* or malay* or nonenglish or norweg* or polish or portug* or roman* or rus* or serb* or slovak or sloven* or spa* or swed* or turk* or ukr* or urdu) not english).lg.
29 22 not (27 or 28)
30 limit 29 to yr = ‘2000–Current’

**Table 4:** Databases and platforms.

Criminal Justice Abstracts	EBSCO
Cumulative Index to Nursing and Allied Health Literature (CINAHL)	EBSCO
International Political Science Abstracts	EBSCO
Scopus	Elsevier
Epistemonikos	Epistemonikos
Australian Criminology Database (CINCH)	Informit
APA PyscINFO	OVID
MEDLINE	OVID
Joanna Briggs Institute EBP Database	OVID
PsycEXTRA	Ovid
Applied Social Sciences Index & Abstracts (ASSIA)	ProQuest
Dissertations & Theses Global	ProQuest
Educational Resources Information Center (ERIC)	ProQuest
International Bibliography of the Social Sciences (IBSS)	ProQuest
Social Services Abstracts	ProQuest
Sociological Abstracts	ProQuest
Conference Proceedings Citation Index – Social Sciences & Humanities (CPCI‐SSH)	Web of Science
Emerging Sources Citation Index (ESCI)	Web of Science
Social Science Citation Index (SSCI)	Web of Science
Book Citation Index – Social Sciences & Humanities	Web of Science

#### 3.2.2 Searching other resources

A number of supplementary sources will be searched by hand, including core journals on terrorism and violent extremism and topics relating to childhood and adolescence (Table 5); grey literature resources (Table 6); and clinical trial repositories (Table 7). When searching journals, all articles published since 2000 (including online first articles) will be reviewed. When searching other websites, the approach used will be tailored to the functionality of the platform, for example, whether it categorises outputs into topics easily identifiable as relevant/irrelevant or allows for detailed searches.

**Table 5:** Key journals.

Journal name
*Terrorism and Political Violence*
*Studies in Conflict & Terrorism*
*Behavioral Sciences of Terrorism and Political Aggression*
*Critical Studies on Terrorism*
*Journal for Deradicalization*
*Perspectives on Terrorism*
*International Journal of Conflict & Violence*
*Dynamics of Asymmetric Conflict*
*Journal of Policing, Intelligence & Counter Terrorism*
*Journal of Threat Assessment and Management*
*Childhood, Children & Society*
*Journal of Child and Family Studies*
*Journal of Youth and Adolescence*
*Vulnerable Children and Youth Studies*
*Journal of Children's Services*
*Child & Family Social Work*

**Table 6:** Grey literature sources.

Source	Link
*Research Centres and Institutes*	
Addressing Violent Extremism and Radicalisation to Terrorism Network (AVERT)	https://www.avert.net.au
Australian Institute of Criminology (AIS)	https://www.aic.gov.au
Centre for Research & Evidence on Security Threats (CREST)	https://crestresearch.ac.uk
Canadian Practitioners Network for the Prevention of Radicalization & Extremist Violence (CPN‐PREV)	https://cpnprev.ca
Danish Institute for International Studies (DIIS)	https://www.diis.dk/en
European Commission Radicalisation Awareness Network (RAN)^a^	https://home-affairs.ec.europa.eu/networks/radicalisation-awareness-network-ran_en
Global Center on Cooperative Security	https://globalcenter.org
Hedayah	https://hedayah.com
Institute for Strategic Dialogue (ISD)	https://www.isdglobal.org
International Centre for Counter‐Terrorism (ICCT)	https://www.icct.nl
International Centre for the Study of Radicalisation (ICSR)	https://icsr.info
International Center for the Study of Violent Extremism (ICSVE)	https://icsve.org
Polarization and Extremism Research and Innovation Lab (PERIL)	https://perilresearch.com
National Consortium for the Study of Terrorism & Responses to Terrorism (START)	https://www.start.umd.edu
PrEval	https://preval.hsfk.de/en/
The PREV‐IMPACT Canada Project	https://prev-impact.ca/en
RAND	https://www.rand.org
Resolve Network	https://www.resolvenet.org
Royal United Services Institute (RUSI)	https://rusi.org
VOX‐Pol	https://voxpol.eu
*Non‐Governmental Organisations*	
Save the Children	https://www.savethechildren.org.uk
Search for Common Ground	https://www.sfcg.org
Violence Prevention Network	https://violence-prevention-network.com
War Child	https://www.warchild.org.uk
Youth Endowment Fund (YEF)	https://youthendowmentfund.org.uk
*Research Repositories*	
Blueprints for Healthy Youth Development	https://www.blueprintsprograms.org
Crime Reduction Toolkit	https://www.college.police.uk/research/crime-reduction-toolkit
CrimeSolutions	https://crimesolutions.ojp.gov
CVE Reference Guide for Local Organizations	https://www.cvereferenceguide.org/en
Radicalisation Research	https://radicalisationresearch.org
*National and International Government Agencies*	
Australian Department of Home Affairs	https://www.homeaffairs.gov.au
Ministry of Foreign Affairs of the Netherlands	https://www.government.nl/ministries/ministry-of-foreign-affairs
Netherlands National Coordinator for Security and Counterterrorism	https://english.nctv.nl
New Zealand Department of the Prime Minister & Cabinet	https://www.dpmc.govt.nz
Organization for Security & Co‐Operation in Europe	https://www.osce.org
Public Safety Canada	https://www.publicsafety.gc.ca/index-en.aspx
Royal Canadian Mounted Police	https://www.rcmp-grc.gc.ca
The Swedish Center for Countering Violent Extremism	https://cve.se
United Nations Educational, Scientific & Cultural Organization	https://www.unesco.org/en
United Nations Development Programme	https://www.undp.org
United Nations Office for Drugs & Crime	https://www.unodc.org
United Nations Office for Counter‐Terrorism	https://www.un.org/counterterrorism/
United Nations Counter‐Terrorism Executive Directorate (UNCTED)	https://www.un.org/securitycouncil/ctc/
United Nations International Children's Emergency Fund (UNICEF)	https://www.unicef.org
USAID	https://www.usaid.gov
US Department of Homeland Security	https://www.dhs.gov
US National Institute of Justice	https://nij.ojp.gov
US Office of Juvenile Justice and Delinquency Prevention	https://ojjdp.ojp.gov
UK Home Office	https://www.gov.uk/government/organisations/home-office
UK Ministry of Justice	https://www.gov.uk/government/organisations/ministry-of-justice

^a^ We will also consult a European Commission report that catalogues EU‐funded projects on preventing radicalisation to identify any websites that appear relevant: European Commission (2022) EU‐funded projects on preventing radicalisation Synergies and Insights.

**Table 7:** Clinical trial registries.

Source
Clinical Trials Results
NIH RePORTER
Trials Register of Promoting Health Interventions (TRoPHI)
Unreported Trials Register
UK Clinical Research Network (UKCRN Study Portfolio)
WHO International Clinical Trials Registry Platform

##### Bibliographic searches

The bibliographies of all relevant, completed Campbell Collaboration systematic reviews conducted through the 5RD countering violent extremism programme will be reviewed,^5^ alongside other relevant review articles (Table 8). This bibliographic analysis will focus only on those studies included in each review.

**Table 8:** Review articles.

Source
Cherney, De Rooy, et al. (2022)
Hassan, Brouillette‐Alarie, Ousman, Kilinc, et al. (2021a)
Hassan, Brouillette‐Alarie, Ousman, Savard, et al. (2021b)
Madriaza et al. (2022)
Morrison et al. (2021)
Pistone et al. (2019)
Wallner (2021)
White (2021)
Wolfowicz et al. (2022)^a^

^a^ Whilst this review specifically focuses on primary interventions, some of those interventions surveyed may also contain elements of secondary prevention.

Relevant reviews include ones completed by members of this research team (Lewis, Marsden, Cherney, et al., 2024), and those by Carthy et al. (2020); Mazerolle, Eggins, et al. (2020); Mazerolle et al. (2021); Sydes et al. (2022); and Windisch et al. (2022). When examining our own review on case management, we will also search through our database of excluded studies using the terms ‘youth’; ‘child’; and ‘adolescent’ to identify any studies that did not report on case management, but which might have reported on another type of youth intervention.

We will also search the bibliographies of high‐quality review articles relating to our phenomenon of interest that have been published in the past 5 years. Whilst our previous review also examined reviews published earlier, recent advances in evaluation practice in the field mean that more recent reviews are more likely to identify relevant studies that might meet our methodological inclusion criteria.

##### Consulting with experts

Following the completion of the main searches, we will consult with up to ten experts researching efforts to counter youth radicalisation to identify additional studies, particularly those that might not be indexed in common platforms. We will also share a preliminary list of studies with members of the 5RD countering violent extremism network to identify any additional studies, particularly those from the grey literature.

##### Forward and backward citation searches

Forward and backward citation searches will be completed for all studies included in the final review following completion of the screening process outlined below. Forward citation searches will be conducted in Google Scholar, and backward citation searches by manually searching the reference lists of included studies. Any potentially eligible studies identified through these searches will be screened using the same process. In the event that any of these additional studies meet our inclusion criteria, they will be included in the review, and subject to the same process of forward and backward citation searches. This process will be repeated until we reach saturation.

### 3.3 Data collection and analysis

#### 3.3.1 Description of methods used in primary research

Extrapolating from previous reviews of CVE interventions (e.g., Lewis, Marsden, Cherney, et al., 2024), it is unlikely that this review will identify any eligible studies using experimental research designs given known challenges facing efforts to deploy these designs in the field of CVE. These include practical challenges relating to small sample sizes, and the potential ethical and legal implications that might result from withholding an intervention for the purposes of evaluating it (Lewis et al., 2020).

Previous Campbell Collaboration reviews of related interventions have highlighted that the use of quasi‐experimental designs remains limited (e.g., Lewis, Marsden, Cherney, et al., 2024; Mazerolle et al., 2021). A small number of authors have used quasi‐experimental research designs to evaluate CVE interventions (Mazerolle et al., 2021), which suggests that this review might identify quasi‐experimental evaluations of relevant interventions. However, a preliminary review of the literature suggests that only a very small number, if any, are likely to meet our definition of a ‘strong’ quasi‐experimental design outlined above. It is therefore likely that a significant proportion of the included studies will be other types of quasi‐experimental or non‐experimental quantitative studies (e.g., cross‐sectional surveys; observational research), and qualitative studies.

One potentially eligible quantitative study comes from Kolbe (2019). This study used secondary data (programme records) collected at baseline, intervention completion (3 months after baseline), and 9 months post‐intervention to examine whether the addition of a home‐based social work component to a group‐based ‘after school enrichment’ programme working with young people in an East African city (aged 13–18 years) explicitly identified as being ‘at risk’ of joining Al‐Shabaab improved levels of programme completion and decreased the likelihood of joining a violent extremist group. The evaluation was made possible by a naturally occurring experiment as the social work component was only introduced after the first cohort had completed the programme and was removed following funding cuts for a fourth and final cohort. Whilst this is one of the few studies identified to date using a control group, analysis of programme documentation is a feature of other quantitative and mixed methods studies of interventions working with at risk or radicalised young people (e.g., Cherney & Belton, 2021). Quantitative analysis of primary data collected from at risk and/or radicalised clients, as well as practitioners and other stakeholders (e.g., through surveys) may also be used to examine the implementation of relevant programmes, for example, when capturing feedback on an intervention or component of an intervention (see Lewis, Marsden, Cherney, et al., 2024). However, this type of data is more commonly captured using qualitative methods, such as focus groups and interviews with clients, practitioners and stakeholders, or observation of practice (see Lewis, Marsden, Cherney, et al., 2024).

#### 3.3.2 Selection of studies

Studies will be screened using a four‐step process:

1. All studies identified through the search strategy above will be imported into EndNote. This initial corpus will be screened for ineligible (e.g., book reviews) and duplicate records before being uploaded into the online systematic review platform Covidence.^6^ Duplicate records will be identified and removed through a) the duplicate detection function included in EndNote and b) a review of all remaining records completed by one trained reviewer.
2. The Covidence platform will automatically screen the uploaded records for any remaining duplicates, and one trained reviewer will review any identified duplicates to ensure that only genuine duplicates are excluded. All remaining records will progress to title and abstract screening.
3. The lead author (JL) will train two reviewers to use the title/abstract screening tool shown in Appendix SI. The trained reviewers will use Covidence to review the first 50 records (sorted on relevance), and their inclusion decisions will be compared to the decisions made by JL to ensure that both reviewers are applying the inclusion criteria correctly and consistently. An online meeting will be held to discuss disagreements, and, if necessary, the exercise repeated.
4. Both reviewers will then screen the remaining abstracts (i.e., abstracts will be screened in duplicate) using this same tool. JL will review 5% of studies excluded by each reviewer to identify any false negatives upon completion of this process, following previous Campbell Collaboration reviews (e.g., Mazerolle, Cherney, et al., 2020). In the event of a high number of false negatives, JL will manually review all records excluded by that reviewer. Once title/abstract screening is completed, all reviewers will meet to discuss any conflicts. In the event that these conflicts cannot be resolved, SM will make a final decision.
5. The same two reviewers will conduct a full text screening of remaining studies in Covidence, guided by a full text screening tool shown in Appendix SI. The same approach outlined above for piloting the screening tool, reviewing exclusion decisions, and resolving any conflicts between reviewers at the title and abstract screening stage will be used.

#### 3.3.3 Data extraction and management

Data extraction will be guided by the data extraction tool presented in Appendix SIII. This tool contains a series of domains to capture bibliographic information about the study (e.g., author(s); title; year and place of publication); details about the intervention being examined (e.g., secondary or tertiary intervention; nature of client base; delivery context; types of practitioner, etc.); methodological information (e.g., type and source of data; research design; sample details, including e.g., whether it includes clients or practitioners; approach to analysis; outcome measures used); data relating to effectiveness and/or implementation (including authors' own conclusions); funding sources and declarations of interest; and the risk of bias as assessed by the reviewers.

All data will be extracted and managed in Microsoft Excel following the data extraction template included as an Appendix. When extracting effect size data relating to effectiveness (Objective 1), effect size data will be independently double‐coded to ensure the accuracy of data inputted into any meta‐analysis.

The conventions for analysing this data are outlined in the sections below.

#### 3.3.4 Assessment of risk of bias in included studies

The risk of bias of studies reporting evidence of effectiveness (Objective 1) will be assessed using either Version 2 of the Cochrane risk‐of‐bias tool for randomised trials (RoB 2) or the Cochrane Risk of Bias in Non‐Randomised Studies – of Interventions (ROBINS‐I) tool (Sterne et al., 2016, 2019). Both tools are organised around a series of domains containing ‘signalling’ questions that will be used to assess the risk of bias present at each domain, and in turn the overall risk of bias (Table 9).

**Table 9:** Risk of bias assessment (Sterne et al., 2016, 2019).

Tool	Domains	Assessment
RoB 2	Bias arising from the randomisation process Bias due to deviation from intervention Bias due to missing outcome data Bias in measurement of outcome Bias in selection of the reported result	High Low Some concerns
ROBINS‐I	*Pre‐Intervention* Bias due to confounding Bias in selection of participants *At‐Intervention* Bias in classification of interventions *Post‐Intervention* Bias due to deviations from interventions Bias due to missing data Bias in measurement of outcome Bias in selection of the reported result	Low Moderate Serious Critical No information

Criteria for determining a study's overall risk of bias are shown in Tables 10 and 11.

Each study will be assessed against the individual domains of the relevant tool shown in Table 9, with these ratings informing an overall risk of bias assessment for each study following the definitions shown in Tables 10 and 11. Assessments for each domain and for a study's overall risk of bias will be presented in summary tables, and sensitivity analysis will be conducted (if possible) to understand the effects of any identified bias.

**Table 10:** Rob 2 risk of bias criteria (Sterne et al., 2019).

Assigned risk level	Description
Low risk of bias	Low risk of bias for all domains
Some concerns	Some concerns for at least one domain but no domain assessed as having a high risk of bias
High risk of bias	High risk of bias for at least one domain; or concerns raised across multiple domains that reduce confidence in the reported results

**Table 11:** ROBINS‐I risk of bias criteria (Sterne et al., 2016).

Assigned risk level	Description
Low risk of bias	Low risk of bias for all domains
Moderate risk of bias	Low or moderate risk of bias for all domains
Serious risk of bias	Serious risk of bias for at least one domain, but no domain has a critical risk of bias
Critical risk of bias	Critical risk of bias for at least one domain

The approach for considering risk of bias within the meta‐analysis will be determined by the number of eligible studies included, and the identified variation in their risk of bias. Following Mazerolle, Cherney, et al. (2020), we may therefore ‘stratify’ the analysis according to different levels of risk or present a singular analysis alongside a ‘narrative discussion of the risk of bias’ (p. 8). The approach taken will be determined by the statistical lead (AS), in consultation with the rest of the research team.

The risk of bias of other types of quantitative (e.g., non‐experimental) studies reporting on implementation (Objectives 2 and 3) will be assessed using the Effective Public Health Practice Project (EPHPP) Quality Assessment Tool for Quantitative Studies, which contains a number of questions relating to selection bias; study design; confounders; blinding; data collection methods; and withdrawals and dropouts.^7^ Qualitative studies, including qualitative components of mixed‐methods studies, will be assessed against a widely used checklist developed by the Critical Appraisal Skills Programme (CASP), which is organised around ten ‘Yes’, ‘No’, or ‘Can't tell’ questions relating to study quality.^8^ Following the conventions set out in Lewis et al. (2023) and Mazerolle, Cherney, et al. (2020), studies for which the answer to the following questions are ‘No’ or ‘Can't Tell’ will be excluded from the review:

– Was the research design appropriate to address the aims of the research?
– Was the recruitment strategy appropriate to the aims of the research?

The results of assessments completed using the CASP and EPHPP tools will be presented in summary tables, and an overall summary of the quality of evidence will be included within the analysis of Objectives 2 and 3.

#### 3.3.5 Measures of treatment effect

Given the focus of the current review on providing evidence for the effectiveness of interventions, only designs that allow for computation of different outcomes between treatments and between treatment and control conditions will be included in the analyses (i.e., RCTs; Cross‐over design; Matched control group design; Unmatched control group designs where the control group has face validity; Unmatched control group designs allowing difference‐in‐difference analysis; Time‐series designs).

For ease of interpretation all effects will be coded such that higher scores would indicate a more positive result (i.e., decrease in support for violent extremism). Some evaluations will likely use continuous measures to report on relevant primary and secondary outcomes (e.g., responses to questions using Likert scales). We will calculate Hedges' *g* (standardised mean differences) for each continuous measure. In the event that evaluations also report on binary outcomes (e.g., whether or not an individual has re‐offended post‐intervention), effect sizes will be computed as odds ratios, and transformed into Hedges' *g* for the meta‐analysis (Borenstein et al., 2021).

Hedge's *g* will be calculated using the formulas described in Borenstein et al. (2021):

g=J×d,

$$V_{g}=J^{2}\times V_{d};\;\mathrm{SE}=\sqrt{V_{g}},$$

where: $J=1-\frac{3}{4\mathrm{df}-1}.$

Similarly, odds ratios will be transformed into standardised mean difference scores (*d*) and then into Hedge's *g* by using the formulas described in Borenstein et al. (2021):

$$d=\mathrm{Log}\;\mathrm{odds}\;\mathrm{ratio}\times \frac{\sqrt{3}}{\pi }.$$

If other inferential tests are reported that allow for transformations to find our desired effects (e.g., *F*, *t*), these transformations will be applied in line with Borenstein et al. (2021).

#### 3.3.6 Unit of analysis issues

It is expected that some unit‐of‐analysis issues will be detected in the data. These will be evaluated on a case‐by‐case basis and suitable methods will be used to ensure the effect sizes that are used in the meta‐analysis are properly calculated. The approaches for addressing issues considered most likely to appear in relevant studies – clustering; multiple intervention groups; and multiple follow‐up periods – are discussed below.

If studies report results for designs with clustering among participants (e.g., assignment of school classes, participants from the same location, or otherwise grouped individuals to treatments), we will adjust for this by inflating standard errors (as described in Higgins et al., 2023, section 23.1.5). Specifically, we will calculate ‘design effect’ using the methods described in Higgins et al. (2023) and use it to adjust the standard error of the effect size estimates. All adjustments will be clearly described in the report for full transparency.

Where a study reports more than one treatment group (i.e., where multiple groups are compared with a single control group), our primary approach would be to avoid double‐counting by splitting the sample size of the shared group (Harrer et al., 2021; Higgins et al., 2019). However, to provide a sensitivity test we will also combine the treatment groups to create a single comparison (i.e., combined treatments vs. control).

If studies produce results on one or more follow‐up periods, our main comparison will be between the baseline and the most immediate follow‐up. However, for exploratory purposes, we will also calculate the effects for the difference between the baseline and the last follow‐up for each study that reports more than one follow‐up to gauge whether the effects hold over time (Higgins et al., 2023, section 6.2.4).

#### 3.3.7 Criteria for determination of independent findings

The review is likely to identify multiple records reporting on the same empirical findings (i.e., the same research study). Any overlap between records will be determined based on an assessment of whether there is any identifiable overlap in authors; research dates and research design; intervention; or references to other included records. In such cases, we will extract and code data from all records meeting our inclusion criteria, but only count individual studies once.

Where two or more overlapping records using an eligible research design present relevant outcome data drawn from the same study, only the record presenting the most complete data will be included in any quantitative analysis. This includes any meta‐analysis or alternative synthesis (see Section 3.3.11). Qualitative data synthesis will include data drawn from all overlapping records, but individual studies will only be included once in presented counts, to avoid overstating the strength of the evidence.

The potential issue of dependency in the data may arise in the likely event that studies report on multiple relevant outcome measures (e.g., studies may present data for two or more outcomes capturing a reduction in support for violent extremism). To address this, we will employ robust variance estimation procedures (Hedges et al., 2010; Pustejovsky & Tipton, 2022; Tipton & Pustejovsky, 2015) that take into account the dependency stemming from collecting multiple measures in the same study (i.e., in an RVE analysis we will include individual measures, not combined effects).

#### 3.3.8 Dealing with missing data

If identified and included studies have missing data, we will take the following actions to address this issue:

1. Search any supplemental materials that may be provided with the study.
2. Reach out to the author(s) to request the missing information (either in the form of providing the missing statistics or the data set from the reported study).
3. Search for other outputs by the author(s) that use the same data.

Studies will be excluded from the meta‐analysis if we are unable to obtain the required information from the authors. However, these studies will still be included in study summaries and in the qualitative assessment, where they present relevant data. When analysing data drawn from studies where a correlation matrix is not available or where not enough information is provided to calculate Hedges *g*, we will calculate the effect sizes for outcomes for which descriptive statistics are sufficiently detailed following Lipsey and Wilson (2001), using the ‘Practical Meta‐Analysis Effect Size Calculator’.

#### 3.3.9 Assessment of heterogeneity

We will assess heterogeneity using both Cochran's Q and the I^2^ statistic (Higgins, 2003) as well as the Tau (Higgins et al., 2023).

#### 3.3.10 Assessment of reporting biases

We will assess publication bias using the following methods:

1. We will use funnel plots with the Egger's Regression Test (Egger et al., 1997) to identify asymmetry between published and non‐published studies and, if possible, examine identified asymmetry through sub‐group analysis.
2. The trim and fill method (Duval & Tweedie, 2000) which makes it possible to check the robustness of the initial findings against publication bias, unless there is high heterogeneity among studies as this can produce unreliable results.
3. If appropriate – that is, if the number of identified studies is greater than 20 and heterogeneity is less than 80% – we will also use the PET‐PEESE method to calculate the precision‐adjusted effect estimate (Stanley, 2017).
4. In the case that heterogeneity of the identified studies is small‐to‐moderate (*I* ^2^ < 50%), we will use the *p*‐curve analysis. This analysis makes it possible to estimate the true effect size and verify whether there is evidence that the intervention effect differs from zero, given the *p*‐values that were obtained in the studies (for heterogeneity >50% such analyses may be performed in subgroups).

#### 3.3.11 Data synthesis

##### Analysis 1: Narrative summary of the evidence base

The first stage of analysis will involve developing a typology of the different types of intervention identified in the research. This typology will be co‐created by JL and JH, and reviewed by SM. Each category covered by the typology will contain interventions that are comparable based on their:

1. Delivery context and/or delivery agents.
2. Target population.
3. Activities.
4. Intended outcomes.

Narrative summaries describing each type of intervention will be presented using the template for intervention description and replication (TIDieR) (Hoffmann et al., 2014). These descriptions will also capture any variations in how specific types of intervention are used, for example, whether case management interventions vary in their intensity. This narrative summary will review the strength of evidence relating to implementation and effectiveness for each type of intervention.

##### Analysis 2: Quantitative analysis of effectiveness data

We will conduct random effects meta‐analyses of relevant primary and secondary outcome measures using data extracted from eligible independent studies using stronger quasi‐experimental and experimental research designs following the conventions for determining independent findings outlined above. Data extracted from studies using treatment versus treatment designs will be synthesised separately from data extracted from studies using treatment versus control/treatment as usual designs, but the conventions for calculating effect sizes will be the same.

We will first combine all measures to assess the overall effect of interventions. We will also investigate and compare the effects of the type of outcome (attitude/intentions/behaviour) using dummy coding in robust variance estimation (as described in Park & Beretvas, 2019). Where two or more studies using treatment versus treatment designs compare the same alternative treatments (based on the typology developed above), we will run a separate meta‐analysis to produce a single effect size.

We will use R to calculate effect sizes and to conduct the meta‐analysis. The metafor package restricted maximum‐likelihood estimator (Viechtbauer, 2010) will be used alongside meta (Balduzzi et al., 2019) and dmetar packages (Harrer et al., 2019). Robust variance estimation will be conducted using the clubSandwich package (Pustejovsky & Tipton, 2022). We will conduct random effects inverse variance meta‐analyses (Lipsey & Wilson, 2001). Mean effect sizes will be reported with confidence intervals, and forest plots for each meta‐analysis will be included.

Where the data allows, the standardised effect sizes for individual studies that cannot be included in the meta‐analysis (i.e., studies that present incomplete data) will be reported. Data drawn from studies that cannot be included in the meta‐analysis will also be synthesised using an alternative method of data synthesis approved by Cochrane (McKenzie & Brennan, 2023). Eligible methods might include summarising effect estimates; combining *p*‐values; and vote counting based on the direction of an observed effect. Any alternative synthesis will be reported in line with the Synthesis Without Meta‐analysis (SWiM) reporting guidelines (Campbell et al., 2020).

##### Analysis 3: Qualitative analysis of implementation data

Data relating to implementation will be synthesised using a framework synthesis approach (Booth & Carroll, 2015) that will use ‘systematic rules or a framework to arrange data into distinct categories that are then synthesised using a variety of techniques such as tables, matrices, and narrative textual summaries’ (Mazerolle, Cherney, et al., 2020, p. 11). This will involve a process of deductive and inductive coding to identify different themes relevant to addressing Objectives 2 and 3. These codes might capture evidence relating to the validity or implementation of different logics, or different implementation factors or moderators found to influence implementation.

We will also be guided by the domains of the RE‐AIM framework when examining implementation factors and moderators, and consider how different factors and moderators affect intervention *Reach* (i.e., its ability to reach the target population, including clients' willingness to engage); perceived intervention *Effectiveness* (as assessed by clients and/or practitioners); intervention *Adoption* (by staff and organisations); the *Implementation* fidelity of an intervention; and the *Maintenance* of an intervention over time (i.e., the extent to which an intervention becomes ‘institutionalised’ within specific settings, as long‐term practice) (Glasgow et al., 2019).

#### 3.3.12 Subgroup analysis and investigation of heterogeneity

Previous reviews of youth CVE interventions highlight the diversity of interventions, and associated implementation factors and moderators, and outcome measures (e.g., White, 2021). Reflecting this diversity, we intend to compare the effects of different interventions and the impact of moderator variables in the analyses by utilising meta‐regression (Borenstein et al., 2021; Harrer et al., 2021). We will use the subgroup correlated effects model (described by Pustejovsky & Tipton, 2022) to conduct moderator analysis for each conceptually similar outcome when an appropriate (ideally at least 10) number of studies for each outcome is identified. For smaller numbers any effects identified could be spurious. Should there be enough studies (i.e., at least 10 that provide us with variables of interest) to warrant such analyses we will assess the impact of the following characteristics of the studies on the relevant effect sizes:

Methodological factors

1. Intervention type (decided based on interventions identified through the review).
2. Type of comparison group (e.g., empty control vs. active control).
3. Methodology (comparing effects found in RCTs vs. quasi‐experimental designs).

Contextual factors

1. Intervention type (decided based on interventions identified through the review).
2. Form of prevention (i.e., secondary vs. tertiary interventions).
3. Intervention length.
4. Location.
5. Delivery context (e.g., clinical, community, etc.).
6. Delivery agents (e.g., clinicians, NGOs, social workers, etc.).
7. Client demographics (e.g., ages, type of extremist ideology, etc.).

The narrative synthesis will also consider differences between findings drawn from studies working with different types of samples as defined in the participant characteristics section, that is, whether the findings specific to working with clients aged 19 years of age or under differ from those drawn from studies examining broader or non‐defined samples of youth.

#### 3.3.13 Sensitivity analysis

In the event that the meta‐analysis contains outcome data drawn from studies reporting on different types of sample (e.g., youth aged 19 years of age or under; youth aged over 19 years of age; youth samples that are not clearly defined) we will conduct sensitivity analysis (i.e., a leave‐one‐out analysis) to examine the impact of including studies reporting on older or non‐defined youth samples on the effect estimates.

Sensitivity analysis will also be performed to examine other differences in study design. For example, we will compare effects found in experimental and quasi‐experimental designs by performing a leave‐one‐out meta‐analysis to assess whether any studies have an undue effect on the findings.

#### 3.3.14 Treatment of qualitative research

Qualitative research will be included when assessing implementation and associated implementation factors and moderators (Objectives 2 and 3) and will be included in the framework synthesis as outlined above. Qualitative data will not be examined in the analysis of effectiveness and will therefore not be included in any quantitative synthesis (i.e., data will not be converted into quantitative metrics).

#### 3.3.15 Summary of findings and assessment of the certainty of the evidence

We do not plan to include Summary of findings and assessment of the certainty of the evidence.

## CONTRIBUTIONS OF AUTHORS

Content: Marsden, Lewis, Stefaniak, Hewitt.

Systematic review methods: Marsden, Lewis, Stefaniak, Hewitt.

Statistical analysis: Stefaniak.

Information retrieval: Marsden, Lewis, Hewitt.

## DECLARATIONS OF INTEREST

No relevant conflicts of interest reported.

## PRELIMINARY TIMEFRAME

We plan to submit the final review by 28th February 2025.

## SOURCES OF SUPPORT

This review is funded by a Campbell Collaboration grant awarded to Sarah Marsden and James Lewis through the Campbell 5RD CVE programme.

## Supporting information

Supporting information.

## Footnotes

1 See https://www.un.org/en/global-issues/youth.

2 See https://www.campbellcollaboration.org/news-and-events/news/new-rfp-countering-violent-extremism-jan-2024.html.

3
https://www.dhs.gov/sites/default/files/2023-07/23_0724_opa_strategic-intelligence-assessment-data-domestic-terrorism.pdf.

4 See https://www.state.gov/foreign-terrorist-organizations/.

5 See https://www.campbellcollaboration.org/research-on-cve.html.

6
https://www.covidence.org.

7 Available at https://www.ephpp.ca/quality-assessment-tool-for-quantitative-studies/.

8 Available at https://casp-uk.net/casp-tools-checklists.
