# Supplementary material for: PROTOCOL: Non‐criminal justice interventions for countering cognitive and behavioural radicalisation amongst children and adolescents: A systematic review of effectiveness and implementation
Source: Campbell Syst Rev. 2025 Jan 15;21(1):e70020. doi: 10.1002/cl2.70020 (PMC11734190; doi:10.1002/cl2.70020)
Supplement: Supplementary file 1 — Supporting information. [file CL2-21-e70020-s001.docx]

**Appendix I. Screening Tools**

Title/ Abstract

| **Topic** | **Inclusion criteria Research Question 1 (Effectiveness)** | **Inclusion criteria Research Questions 2 & 3 (Implementation)** | **Exclusion criteria** |
| --- | --- | --- | --- |
| Duplicate | N/A | | Duplicate title, secondary title, author, year to be excluded. |
| Topic | Study focuses on efforts to prevent and counter cognitive and/or behavioural forms of radicalisation | | Study does focus on efforts to counter cognitive and/or behavioural forms of radicalisation |
| Year of Publication | 2000 onwards | | Published before 2000 |
| Language | English | | Language other than English |

Full Text

| **Topic** | **Inclusion criteria Research Question 1 (Effectiveness)** | | **Inclusion criteria Research Questions 2 & 3 (Implementation)** | **Exclusion criteria** |
| --- | --- | --- | --- | --- |
| Duplicate | N/A | | | Duplicate record published in another format (e.g., journal article & chapter in PhD thesis) |
| Research design | Quantitative study using an experimental or stronger quasi-experimental research design to report on primary or secondary research data.  Eligible designs include:  • Randomised controlled trials  • Cross‐over designs  • Propensity or statistically matched control group designs (with or without baseline)  • Unmatched control group designs without baseline where control group has face validity  • Unmatched control group designs with pre-post intervention measures allowing for difference‐in‐difference analysis.  • Short interrupted time‐series designs with control group (less than 25 pre-and post-intervention observations)  • Long interrupted time-series designs with or without a control group (over 25 pre-and post-intervention observations) | | Quantitative study using an experimental, quasi-experimental or non-experimental research design to report on primary or secondary data.  OR  Qualitative study using any research design to report on primary or secondary data. | Study does not meet inclusion criteria Q1-3. |
| Population (I) | Intervention works with children and adolescents aged 0-19 (or with members of their familial or social network).  Secondary or tertiary intervention working with at-risk (secondary) or radicalised (tertiary) children and adolescents. | | | Intervention does not work with children and adolescents (or familial or social networks)  Primary intervention working with broader populations of children and adolescents |
| Population (II) | Reports on data specifically relating to working with children and adolescents aged 0-19 (i.e., reports on an intervention that only works with this age group, or presents data drawn from sub-set of clients who are aged 19 or under) | At least 50 per cent of client sample is aged 19 years or under | | Data is not specific to work with children or adolescents. |
| Intervention | Intervention operates outside of the criminal justice system:  1. Work with individuals before they formally come into contact with the criminal justice system.  2. Provided as an alternative to arrest, charge, or imprisonment to individuals who are/ have previously been subject to a criminal investigation or arrest.  3. Work with individuals who have previously been or are subject to a criminal investigation, arrest, prosecution, and/ or sentence but which operate independently from criminal justice agencies, or the criminal justice system. | | | Intervention delivered in and through the criminal justice system (e.g., in correctional or probation contexts) |
| Comparator | No restrictions | | | N/A |
| Problem | Interventions explicitly focus on countering cognitive and/or behavioural radicalisation. | | | Not explicitly focused on tackling cognitive or behavioural radicalisation |
| Outcomes | Study reports on primary or secondary outcomes relevant to countering cognitive or behavioural radicalisation.  *Primary outcomes*  Attitudes (e.g., reduction in support or sympathy for a violent cause).  Intentions (e.g., reducing an individual's willingness to engage in violent action).  Behaviours (e.g., disengagement or desistance from violent extremism).  *Secondary outcomes*  Dynamic risk factors linked to cognitive or behavioural radicalisation (e.g., socio-demographic; attitudinal; psychological/ personality; experiential'; criminogenic; personal; push; pull) | | Study examines how an intervention is being implemented.  And/or  Study reports on implementation factors (facilitators, barriers) and moderators relevant to implementation. | No discussion of either element. |

**Appendix II. Quality Assessment Tools**

|  | | | | |
| --- | --- | --- | --- | --- |
| **Risk of Bias: Non-Randomised Studies: ROBINS-I^[[1]](#footnote-2)^** | | | | |
| **ROBINS-I (I)**  **Bias due to Confounding** | | 1.1. Is there potential for confounding of the effect of intervention in this study? | Yes  Probably Yes  Probably No  No  *If No/ Probably No, risk of bias is low for this domain and no further signalling questions are required..* | |
|  |  | *Only if Yes/ Probably Yes at 1.1.*  1.2. Was the analysis based on splitting participants’ follow up time according to intervention received? | N/A  Yes  Probably Yes  Probably No  No  No Information | |
|  |  | *Only if Yes/ Probably Yes at 1.2*  1.3. Were intervention discontinuations or switches likely to be related to factors that are prognostic for the outcome? | N/A  Yes  Probably Yes  Probably No  No  No Information | |
|  |  | *Only if Yes/ Probably Yes at 1.1.*  1.4. Did the authors use an appropriate analysis method that controlled for all the important confounding areas? | N/A  Yes  Probably Yes  Probably No  No  No Information | |
|  |  | *Only if Yes/ Probably Yes at 1.4.*  1.5. Were confounding areas that were controlled for measured validly and reliably by the variables available in this study? | N/A  Yes  Probably Yes  Probably No  No  No Information | |
|  |  | *Only if Yes/ Probably Yes at 1.1.*  1.6. Did the authors control for any post-intervention variables? | N/A  Yes  Probably Yes  Probably No  No  No Information | |
|  |  | *Only if Yes/ Probably Yes at 1.3*  1.7. Did the authors use an appropriate analysis method that adjusted for all the important confounding areas and for time varying confounding? | N/A  Yes  Probably Yes  Probably No  No  No Information | |
|  |  | *If Yes/ Probably Yes at 1.7*  1.8. Were confounding areas that were adjusted for measured validly and reliably by the variables available in this study? | N/A  Yes  Probably Yes  Probably No  No  No Information | |
|  |  | Risk of Bias for Domain | Low  Moderate  Serious  Critical  No Information | |
| **ROBINS-I (II)**  **2. Bias in selection of participants into the study** | | 2.1. Was selection of participants into the study (or into the analysis) based on participant characteristics observed after the start of intervention? | Yes  Probably Yes  Probably No (Go to 2.4)  No (Go to 2.4)  No Information | |
|  |  | *Only if Yes/ Probably Yes at 2.1*  2.2. Were the post-intervention variables that influenced selection likely to be associated with intervention? | N/A  Yes  Probably Yes  Probably No  No  No Information | |
|  |  | *If Yes/ Probably Yes at 2.2*  2.3. Were the post-intervention variables that influenced selection likely to be influenced by the outcome or a cause of the outcome? | N/A  Yes  Probably Yes  Probably No  No  No Information | |
|  |  | 2.4. Do start of follow-up and start of intervention coincide for most participants? | Yes  Probably Yes  Probably No  No  No Information | |
|  |  | *If Yes /Probably Yes to 2.2 & 2.3, or*  *If No/ Probably No to 2.4*  2.5. Were adjustment techniques used that are likely to correct for the presence of selection biases? | N/A  Yes  Probably Yes  Probably No  No  No Information | |
|  |  | Risk of Bias Judgement for Domain | Low  Moderate  Serious  Critical  No Information | |
| **ROBINS-I (III)**  **3. Bias in classification of interventions** | | 3.1. Were intervention groups clearly defined? | Yes  Probably Yes  Probably No  No  No Information | |
|  |  | 3.2. Was the information used to define intervention groups recorded at the start of the intervention? | Yes  Probably Yes  Probably No  No  No Information | |
|  |  | 3.3. Could classification of intervention status have been affected by knowledge of the outcome or risk of the outcome? | Yes  Probably Yes  Probably No  No  No Information | |
|  |  | Risk of Bias for Domain | Low  Moderate  Serious  Critical  No Information | |
| **ROBINS-I (IV)**  **4. Bias as a result of departures from intended interventions** | | 4.1. Were there deviations from the intended intervention beyond what would be expected in usual practice? | Yes  Probably Yes  Probably No  No  No Information | |
|  |  | *If Yes/ Probably Yes to 4.1.*  4.2. Were these deviations from intended intervention unbalanced between groups and likely to have affected the outcome? | N/A  Yes  Probably Yes  Probably No  No  No Information | |
|  |  | 4.3. Were important cointerventions balanced across intervention groups? | Yes  Probably Yes  Probably No  No  No Information | |
|  |  | 4.4. Was the intervention implemented successfully for most participants? | Yes  Probably Yes  Probably No  No  No Information | |
|  |  | 4.5. Did study participants adhere to the assigned intervention regimen? | Yes  Probably Yes  Probably No  No  No Information | |
|  |  | *If No/ Probably No to 4.3, 4.4 or 4.5.*  4.6. Was an appropriate analysis used to estimate the effect of starting and adhering to the intervention? | N/A  Yes  Probably Yes  Probably No  No  No Information | |
|  |  | Risk of Bias Judgement for Domain | Low  Moderate  Serious  Critical  No Information | |
| **ROBINS-I (V)**  **5. Bias as a result of missing data** | | 5.1. Were outcome data available for all, or nearly all, participants? | Yes  Probably Yes  Probably No  No  No Information | |
|  |  | 5.2. Were participants excluded due to missing data on intervention status? | Yes  Probably Yes  Probably No  No  No Information | |
|  |  | 5.3. Were participants excluded due to missing data on other variables needed for the analysis? | Yes  Probably Yes  Probably No  No  No Information | |
|  |  | *If No/ Probably No to 5.1 or*  *If Yes/ Probably Yes to 5.2 or 5.3*  5.4. Are the proportion of participants and reasons for missing data similar across interventions? | N/A  Yes  Probably Yes  Probably No  No  No Information | |
|  |  | *If No/ Probably No to 5.1 or*  *If Yes/ Probably Yes to 5.2 or 5.3*  5.5. Is there evidence that results were robust to the presence of missing data? | N/A  Yes  Probably Yes  Probably No  No  No Information | |
|  |  | Risk of Bias Judgement for Domain | Low  Moderate  Serious  Critical  No Information | |
| **ROBINS-I (VI)**  **6. Bias in measurement of outcomes** | | 6.1. Could the outcome measure have been influenced by knowledge of the intervention received? | Yes  Probably Yes  Probably No  No  No Information | |
|  |  | 6.2. Were outcome assessors aware of the intervention received by study participants? | Yes  Probably Yes  Probably No  No  No Information | |
|  |  | 6.3. Were the methods of outcome assessment comparable across intervention groups? | Yes  Probably Yes  Probably No  No  No Information | |
|  |  | 6.4. Were any systematic errors in measurement of the outcome related to intervention received? | Yes  Probably Yes  Probably No  No  No Information | |
|  |  | Risk of Bias Judgement for Domain | Low  Moderate  Serious  Critical  No Information | |
| **ROBINS-I (VII)**  **7. Bias in selection of the reported result** | | 7.1. Is the reported effect estimate likely to be selected, on the basis of the results, from multiple outcome measurements within the outcome domain? | Yes  Probably Yes  Probably No  No  No Information | |
|  |  | 7.2. Is the reported effect estimate likely to be selected, on the basis of the results, from multiple analyses of the intervention–outcome relationship? | Yes  Probably Yes  Probably No  No  No Information | |
|  |  | 7.3. Is the reported effect estimate likely to be selected, on the basis of the results, from different subgroups? | Yes  Probably Yes  Probably No  No  No Information | |
|  |  | Risk of Bias Judgement for Domain | Low  Moderate  Serious  Critical  No Information | |
| **Overall Risk of Bias** | | Overall risk of bias | Low  Moderate  Serious  Critical | |
| **Risk of Bias: Randomised Studies: RoB 2^[[2]](#footnote-3)^** | | | | |
| **ROB 2 (I)**  **Bias arising from the randomisation process** | 1.1 Was the allocation sequence random? | | | Yes  Probably Yes  Probably No  No  No Information |
|  | 1.2 Was the allocation sequence concealed until participants were enrolled and assigned to interventions? | | | Yes  Probably Yes  Probably No  No  No Information |
|  | 1.3 Did baseline differences between intervention groups suggest a problem with the randomisation process? | | | Yes  Probably Yes  Probably No  No  No Information |
|  | Risk of Bias for Domain | | | High  Low  Some Concerns |
| **ROB 2 (II)**  **Bias due to deviations from intended interventions** | 2.1 Were participants aware of their assigned intervention during the trial? | | | Yes  Probably Yes  Probably No  No  No Information |
|  | 2.2 Were carers and people delivering the interventions aware of participants’ assigned intervention during the trial? | | | Yes  Probably Yes  Probably No  No  No Information |
|  | If Yes/ Probably Yes /No Information to 2.1 or 2.2  2.3. Were there deviations from the intended intervention that arose because of the trial context? | | | N/A  Yes  Probably Yes  Probably No  No  No Information |
|  | If Yes/ Probably Yes to 2.3  2.4. Were these deviations likely to have affected the outcome? | | | N/A  Yes  Probably Yes  Probably No  No  No Information |
|  | If Yes/ Probably Yes/ No Information to 2.4.  2.5. Were these deviations from intended intervention balanced between groups? | | | N/A  Yes  Probably Yes  Probably No  No  No Information |
|  | 2.6 Was an appropriate analysis used to estimate the effect of assignment to intervention? | | | Yes  Probably Yes  Probably No  No  No Information |
|  | If No /Probably No/ No Information to 2.6.  2.7 Was there potential for a substantial impact (on the result) of the failure to analyse participants in the group to which they were randomised? | | | N/A  Yes  Probably Yes  Probably No  No  No Information |
|  | Risk of Bias for Domain | | | High  Low  Some Concerns |
| **RoB 2 (III)**  **Bias due to missing outcome data** | 3.1 Were data for this outcome available for all, or nearly all, participants randomised? | | | Yes  Probably Yes  Probably No  No  No Information |
|  | If No /Probably No /No Information to 3.1.  3.2 Is there evidence that the result was not biased by missing outcome data? | | | N/A  Yes  Probably Yes  Probably No  No |
|  | If No /Probably No to 3.2.  3.3 Could missingness in the outcome depend on its true value? | | | N/A  Yes  Probably Yes  Probably No  No  No Information |
|  | If Yes/ Probably Yes /No Information to 3.3.  3.4 Is it likely that missingness in the outcome depended on its true value? | | | N/A  Yes  Probably Yes  Probably No  No  No Information |
|  | Risk of Bias for Domain | | | High  Low  Some Concerns |
| **RoB 2 (IV)**  **Bias in measurement of outcome** | 4.1 Was the method of measuring the outcome inappropriate? | | | Yes  Probably Yes  Probably No  No  No Information |
|  | 4.2 Could measurement or ascertainment of the outcome have differed between intervention groups? | | | Yes  Probably Yes  Probably No  No  No Information |
|  | If No /Probably No / No Information to 4.1 & 4.2.  4.3 Were outcome assessors aware of the intervention received by study participants? | | | Yes  Probably Yes  Probably No  No  No Information |
|  | If Yes /Probably Yes/ No Information to 4.3.  4.4 Could assessment of the outcome have been influenced by knowledge of intervention received? | | | N/A  Yes  Probably Yes  Probably No  No  No Information |
|  | If Yes /Probably Yes/ No Information to 4.4.  4.5 Is it likely that assessment of the outcome was influenced by knowledge of intervention received? | | | N/A  Yes  Probably Yes  Probably No  No  No Information |
|  | Risk of Bias for Domain | | | High  Low  Some Concerns |
| **RoB 2 (V)**  **Bias in selection of the reported result** | 5.1 Were the data that produced this result analysed in accordance with a prespecified analysis plan that was finalised before unblinded outcome data were available for analysis? | | | Yes  Probably Yes  Probably No  No  No Information |
|  | 5.2 . Is the numerical result being assessed likely to have been selected, on the basis of the results, from: multiple eligible outcome measurements (eg, scales, definitions, time points) within the outcome domain? | | | Yes  Probably Yes  Probably No  No  No Information |
|  | 5.3 . Is the numerical result being assessed likely to have been selected, on the basis of the results, from: multiple eligible analyses of the data? | | | Yes  Probably Yes  Probably No  No  No Information |
|  | Risk of Bias Judgement for Domain | | | Low  Moderate  Serious  Critical  No Information |
| **Overall Risk of Bias** | Risk of Bias | | | High  Low  Some Concerns |

| **Risk of Bias: EPHPP Quality Assessment Tool^[[3]](#footnote-4)^** | | |
| --- | --- | --- |
| **A Selection Bias** | Are the individuals selected to participate in the study likely to be representative of the target population? | 1 Very likely  2 Somewhat likely  3 Not likely  4 Can’t tell |
|  | What percentage of selected individuals agreed to participate? | 1 80 - 100%  2 60 – 79%  3 less than 60%  4 Not applicable  5 Can’t tell |
|  | Overall rating for section | 1 Strong  2 Moderate  3 Weak |
| **B Study Design** | Indicate the study design | 1 Randomized controlled trial  2 Controlled clinical trial  3 Cohort analytic (two group pre + post)  4 Case-control  5 Cohort (one group pre + post (before & after))  6 Interrupted time series  7 Other (specify) |
|  | Was the study described as randomized? | 1 No (Go to component C)  2 Yes |
|  | If Yes  Was the method of randomization described? | 1 No  2 Yes |
|  | If Yes  Was the method appropriate? | 1 No  2 Yes |
|  | Overall rating for section | 1 Strong  2 Moderate  3 Weak |
| **C CONFOUNDERS** | Were there important differences between groups prior to the intervention?  Examples of confounders:  1 Race; 2 Sex; 3 Marital status/family; 4 Age;  5 SES (income or class); 6 Education; 7 Health status; 8 Pre-intervention score on outcome measure. | 1 Yes  2 No  3 Can’t tell |
|  | If Yes.  Indicate the percentage of relevant confounders that were controlled (either in the design (e.g. stratification, matching) or analysis)? | 1 80 – 100% (most)  2 60 – 79% (some)  3 Less than 60% (few or none)  4 Can’t Tell |
|  | Overall rating for section | 1 Strong  2 Moderate  3 Weak |
| **D BLINDING** | Was (were) the outcome assessor(s) aware of the intervention or exposure status of participants? | 1 Yes  2 No  3 Can’t tell |
|  | Were the study participants aware of the research question? | 1 Yes  2 No  3 Can’t tell |
|  | Overall rating for section | 1 Strong  2 Moderate  3 Weak |
| **E DATA COLLECTION METHODS** | Were data collection tools shown to be valid? | 1 Yes  2 No  3 Can’t tell |
|  | Were data collection tools shown to be reliable? | 1 Yes  2 No  3 Can’t tell |
|  | Overall rating for section | 1 Strong  2 Moderate  3 Weak |
| **F WITHDRAWALS AND DROP- OUTS** | Were withdrawals and drop-outs reported in terms of numbers and/or reasons per group? | 1 Yes  2 No  3 Can’t tell  4 Not Applicable (i.e. one time surveys or interviews) |
|  | Indicate the percentage of participants completing the study. (If the percentage differs by groups, record the lowest). | 1 80 -100%  2 60-79%  3 less than 60%  4 Can’t tell  5 Not Applicable (i.e. Retrospective case-control) |
|  | Overall rating for section | 1 Strong  2 Moderate  3 Weak  Not applicable |
| **G INTERVENTION INTEGRITY** | What percentage of participants received the allocated intervention or exposure of interest | 1 80 -100%  2 60-79%  3 less than 60%  4 Can’t tell |
|  | Was the consistency of the intervention measured? | 1 Yes  2 No  3 Can’t tell |
|  | Is it likely that the subjects received an unintended intervention (contamination or co-intervention) that may the results? | 1 Yes  2 No  3 Can’t tell |
| **H ANALYSES** | Indicate the unit of allocation (single code) | Community  Organization/institution  Practice/office  Individual |
|  | Indicate the unit of analysis (single code) | Community  Organization/institution  Practice/office  Individual |
|  | Are the statistical methods appropriate for the study design? | 1 Yes  2 No  3 Can’t tell |
|  | Is the analysis performed by intervention allocation status (i.e. intention to treat) rather than the actual intervention received? | 1 Yes  2 No  3 Can’t tell |
| **GLOBAL RATING** | Overall quality | 1 STRONG (No WEAK ratings)  2 MODERATE (One WEAK rating)  3 WEAK (Two or more WEAK ratings) |

| **Assessment of Qualitative Research CASP Checklist^[[4]](#footnote-5)^** | | |
| --- | --- | --- |
| Question | Answer Options | Consider |
| Was there a clear statement of the aims of the research? | Yes  No  Can’t Tell | - What was the goal of the research; - Why it was thought important; - Its relevance. |
| Is a qualitative methodology appropriate? | Yes  No  Can’t Tell | - If the research seeks to interpret or illuminate the actions and/or subjective experiences of research participants; - Is qualitative research the right methodology for addressing the research goal. |
| Was the research design appropriate to address the aims of the research? | Yes  No  Can’t Tell | - If the researcher has justified the research design (e.g. have they discussed how they decided which method to use) |
| Was the recruitment strategy appropriate to the aims of the research? | Yes  No  Can’t Tell | - If the researcher has explained how the participants were selected; - If they explained why the participants they selected were the most appropriate to provide access to the type of knowledge sought by the study; - If there are any discussions around recruitment (e.g. why some people chose not to take part) |
| Was the data collected in a way that addressed the research issue? | Yes  No  Can’t Tell | - If the setting for the data collection was justified; - If it is clear how data were collected (e.g. focus group, semi-structured interview etc.); - If the researcher has justified the methods chosen; - If the researcher has made the methods explicit (e.g. for interview method, is there an indication of how interviews are conducted, or did they use a topic guide); - If methods were modified during the study. If so, has the researcher explained how and why; - If the form of data is clear (e.g. tape recordings, video material, notes etc.); - If the researcher has discussed saturation of data |
| Has the relationship between researcher and participants been adequately considered? | Yes  No  Can’t Tell | - If the researcher critically examined their own role, potential bias and influence during (a) formulation of the research questions (b) data collection, including sample recruitment and choice of location; - How the researcher responded to events during the study and whether they considered the implications of any changes in the research design. |
| Have ethical issues been taken into consideration? | Yes  No  Can’t Tell | - If there are sufficient details of how the research was explained to participants for the reader to assess whether ethical standards were maintained; - If the researcher has discussed issues raised by the study (e.g. issues around informed consent or confidentiality or how they have handled the effects of the study on the participants during and after the study); - If approval has been sought from the ethics committee. |
| Was the data analysis sufficiently rigorous? | Yes  No  Can’t Tell | - If there is an in-depth description of the analysis process; - If thematic analysis is used. If so, is it clear how the categories/themes were derived from the data; - Whether the researcher explains how the data presented were selected from the original sample to demonstrate the analysis process; - If sufficient data are presented to support the findings; - To what extent contradictory data are taken into account; - Whether the researcher critically examined their own role, potential bias and influence during analysis and selection of data for presentation. |
| Is there a clear statement of findings? | Yes  No  Can’t Tell | - If the findings are explicit; - If there is adequate discussion of the evidence both for and against the researcher’s arguments; - If the researcher has discussed the credibility of their findings (e.g. triangulation, respondent validation, more than one analyst); - If the findings are discussed in relation to the original research question |
| How valuable is the research? | Free text. | - If the researcher discusses the contribution the study makes to existing knowledge. - If they identify new areas where research is necessary - If the researchers have discussed whether or how the findings can be transferred to other populations or considered other ways the research may be used.   Note: For review purposes, we converted free text for this question into a yes/ no answer to the question of whether the research was valuable based on this criteria. |

**Appendix III. Data Extraction Tool**

| **Category** | **Field** | | **Type** |
| --- | --- | --- | --- |
| **Record**  **Information** | Author | | Free text |
|  | Year | | Free text |
|  | Title | | Free text |
|  | Bibliographic information | | Free text |
|  | Record type | | Pre-defined codes (single code)   - Journal article - Book - Book chapter - Research institution report - Government/ public agency report - Dissertation/ thesis - Other (specify) |
|  | Publication status | | Pre-defined codes (single code)   - Published - Unpublished |
|  | Overview of study | | Free text |
|  | Funding source | | Free text |
|  | Any declaration of interest | | Free text |
| **Research Design** | Type of research | | Pre-defined codes (single code)   - Quantitative - Qualitative - Mixed methods |
|  | Quantitative design  (if relevant) | | Pre-defined codes (single code)   - Experimental (specify) - Strong quasi-experimental (specify) - Weaker quasi experimental (specify) - Other (specify) |
|  | Comparator condition  (if relevant) | | Pre-defined codes (single code)   - Treatment as usual - Alternative treatment (specify) - No treatment - Waitlist |
|  | Type of comparison  (if relevant) | | Specify (e.g., empty vs. active; comparison between two interventions; comparison between versions of same intervention, etc.) |
|  | Randomisation  (if relevant) | | Pre-defined codes (single code)   - Randomised - Quasi-randomised - Non-randomised - Other (specify) |
|  | Qualitative design  (if relevant) | | Free text |
| **Data Collection** | Quantitative data  (if relevant) | | Free text - specify:  - Primary or secondary data  - Type/ form of data  - Data collection tools |
|  | Qualitative data  (if relevant) | | Free text - specify  - Primary or secondary data  - Type/ form of data  - Data collection tools |
|  | Data source/ sample | | Pre-defined codes (multi-code if needed)   - Practitioners (specify) - Other stakeholders (specify) - Children and/or adolescents - Family/ social networks (specify) - Other (specify) |
|  | Sample size | | Free text - specify sample size for each data source and data type listed above. |
| **Data Analysis** | Sample description | | Free text - sample as described in study |
|  | Approach to data analysis | | Free text - analysis as described in study |
|  | Treatment of data relating to children and adolescents. | | Pre-defined codes (single code)  - All data relates to work with 0-19s  - Subgroup analysis of work with 0-19s  - Data relating to youth broadly defined |
|  | Research question eligibility | | Pre-defined codes (multi-code if needed)   - Q1. Effectiveness - Q2. Implemented as intended - Q3. Implementation factors /moderators |
| **Intervention Details^[[5]](#footnote-6)^** | Intervention name | | Free text |
|  | Intervention description | | Free text |
|  | Country of delivery | | Free text |
|  | Age of clients (note, specify age of index client in event that programme works with families or peers only i.e. the person who is at risk or radicalised) | | Pre-defined codes (multi-code if needed)  - 0-9 years old  - 10-14 years old  - 15-19 years old  - 20-24 years old  - Over 25 years old |
|  | Other relevant client demographic information (e.g., types of ideology, etc.) | | Free text |
|  | Prevention | | Pre-defined codes (multi-code if needed)   - Secondary prevention   - Tertiary prevention |
|  | Contact with CJS | | Pre-defined codes (single code)  -No contact with CJS (i.e. pre-crime)  - Diversion from CJS  - Pre-arrest (specify)  - Pre-prosecution (specify)  - Pre-sentencing (specify) |
|  | Direct or Indirect Prevention | | Pre-defined codes (single code))  - Works directly with youth  - Works indirectly through families/ peers  - Combines direct and indirect work |
|  | Intervention length | | Free text |
| **Intervention Context** | Context | | Free text - brief description of delivery context (e.g., community, clinical, educational etc.) and any implementation factors and moderators as defined above. |
|  | Delivery agents | | Free text - description of delivery agents |
|  | Criminal justice agencies | | Pre-defined codes (single code)  - Criminal justice agencies involved (specify)  - Criminal justice agencies not involved. |
| **Data Analysis - Research Question 1 (Effectiveness)** | | | |
| **Progress and Outcome Measures**  **(Study Level)** | Is effectiveness assessed using eligible research design? | | Pre-defined codes (single code)  -Yes  -No |
|  | How is effectiveness assessed? | | Free text |
|  | Primary outcomes | | List of all primary outcomes in study. |
|  | Secondary outcomes | | List of all secondary outcomes in study. |
|  | Author's overall conclusions on intervention effectiveness | | Free text - summary of conclusions. |
|  | Coder’s overall assessment of intervention effectiveness | | Pre-defined codes   - Effective (specify) - Promising (specify) - Mixed results (specify) - Ineffective (specify)   - Unclear (specify) |
| **Outcome Data^[[6]](#footnote-7)^**  **(Separate Row for Each Primary/ Secondary outcome)** | Study Details | | Full reference for study |
|  | Outcome | | Free text - measure and description. |
|  | Outcome type | | Pre-defined codes (single code)   - Primary outcome - Secondary outcome |
|  | Primary outcome type  (if relevant) | | Pre-defined codes (single code)  - Attitudes  - Intentions  - Behaviours |
|  | Secondary outcome type  (if relevant) | | Pre-defined codes (single code)  - Socio-demographic/ background  - Attitudinal  - Psychological/ Personality  - Experiential  - Criminogenic |
|  | Measurement tool | | Free text – name/ type of tool used to capture primary/ secondary outcome. |
|  | Source of data used in original analysis. | | Pre-defined codes   - Self-report - Observation - Official source (specify) - Interview - Other (specify) |
|  | Psychometric properties of the measurement tool (e.g., reliability, validity, etc.)? | | Free text – description of properties. |
|  | Type of respondent/ data source. | | Free text – description of the type of respondent from which data was collected (e.g. client, practitioner etc.) |
|  | Time-points at which measurement taken. | | Free text - time-points at which measure collected (e.g. pre/ post intervention) |
|  | Short or delayed effect | | Immediate measurement of outcome  Delayed measurement of outcome |
|  | Was data collected in same way for treatment and comparison condition? | | Pre-defined codes (single code)   - Yes - No (specify) - Unclear |
|  | Are there any raw differences (i.e. significant or non-significant) differences between control and treatment group? | | Pre-defined codes (single code)   - Yes – results favour treatment - Yes – results favour comparison - No - Unclear |
|  | Direction of outcome change | | Pre-defined codes (single code)   - Positive - Negative - Mixed (specify) - Unclear |
|  | Statistically significant differences for outcome | | Pre-defined codes (single code)   - Yes - No - Not tested - Unclear |
|  | Study author(s)’ conclusions | | Free text – outline conclusions drawn in original study about this outcome. |
|  | Coder’s conclusions | | Free text |
| **Effect Size^[[7]](#footnote-8)^**  **(Separate Row for Each Effect Size)** | Page number | | Free text – page number on which effect size reported. |
|  | Type of effect captured | | Pre-defined codes (single code)   - Post-intervention only - Pre-intervention & post-intervention   - Follow-up after initial post-intervention measurement. |
|  | Timeframe captured | | Free text for all options.   - Minimum time - Maximum time - Mean time - Fixed (single code) |
|  | How effect size is captured | | Pre-defined codes (single code)   - Reported in document - Calculated by research team |
|  | *If reported in document*  Effect size | | Free text – reported effect size. |
|  | *If calculated by researchers*  Data and calculations used to calculate effect size | | Free text – data used, calculations and final effect size calculated. |
| **Data Analysis - Research Question 2 & 3 (Implementation)** | | | |
| **Q2 – Process of Implementation** | | Is the process of implementation assessed? | Pre-defined codes (single code)  -Yes  -No |
|  |  | How is process of implementation assessed? | Free text |
|  |  | Source of data used in original analysis. | Pre-defined codes   - Self-report - Observation - Official source (specify) - Interview - Other (specify) |
|  |  | Type of respondent/ data source. | Free text – description of the type of respondent from which data was collected (e.g. client, practitioner etc.) |
|  |  | Implemented in way expected? | Free text – description of extent to which implemented in way expected |
|  |  | Evidence | Free text – positive research findings (page number) |
|  |  | Evidence opposed to specific intervention, tool, or approach | Free text – negative research findings (page number) |
|  |  | Study author(s)’ conclusions | Free text – outline conclusions drawn in original study about process. |
|  |  | Coder’s conclusions | Free text –coder conclusions about process of implementation. |
| **Q3 – Implementation Factors/ Moderators** | | Implementation factors/ moderators. | Free text – describe factors/ moderators discussed. |
|  |  | Source of data used in original analysis. | Pre-defined codes   - Self-report - Observation - Official source - Interview   - Other (specify) |
|  |  | Type of respondent. | Free text – description of the type of respondent from which data was collected (e.g. client, practitioner etc.) |
|  |  | Evidence of implementation factors/ moderators having a positive effect on process.  (Separate row for each factor or moderator) | Free text – positive findings |
|  |  | Evidence of implementation factors/ moderators having a negative effect on process.  (Separate row for each factor or moderator) | Free text – negative findings |
|  |  | Study author(s)’ conclusions | Free text – outline conclusions drawn in original study about factor(s) |
|  |  | Coder’s conclusions | Free text –coder conclusions |

1. Based on Sterne, J. A. C., Higgins, J. P. T., Elbers, R. G. , Reeves, B. C. and the development group for ROBINS- I. Risk Of Bias In Non-randomized Studies of Interventions (ROBINS-I): detailed guidance, updated 12 October 2016. Available from http://www.riskofbias.info [accessed 12^th^ April 2022]. [↑](#footnote-ref-2)
2. Based on Sterne, J. A. C., Savović, J., Page, M. J., Elbers, R. G., Blencowe, N. S., Boutron, I., Cates, C. J., Cheng, H-Y., Corbett, M. S., Eldridge, S. M., Hernán, M. A., Hopewell, S., Hróbjartsson, A., Junqueira, D. R., Jüni, P., Kirkham, J. J., Lasserson, T., Li, T., McAleenan, A., Reeves, B. C., Shepperd, S., Shrier, I., Stewart, L. A., Tilling, K., White, I. R., Whiting, P. F., & Higgins, J. P. T. (2019) RoB 2: a revised tool for assessing risk of bias in randomised trials. BMJ, 366, l4898 [↑](#footnote-ref-3)
3. https://www.ephpp.ca/PDF/Quality%20Assessment%20Tool_2010_2.pdf [↑](#footnote-ref-4)
4. Available at https://casp-uk.net/casp-tools-checklists/. The original tool also includes a question relating to how the value of the research that was not used to assess studies. [↑](#footnote-ref-5)
5. Separate row for each intervention examined in individual study. [↑](#footnote-ref-6)
6. Based on coding tool first developed by Mazerolle et al. (2020) as used in Lewis et al. (2023) [↑](#footnote-ref-7)
7. Based on coding tool first developed by Mazerolle et al. (2020) as used in Lewis et al. (2023). [↑](#footnote-ref-8)
